# *WRINKLED1*, A Ubiquitous Regulator in Oil Accumulating Tissues from *Arabidopsis* Embryos to Oil Palm Mesocarp

**DOI:** 10.1371/journal.pone.0068887

**Published:** 2013-07-26

**Authors:** Wei Ma, Que Kong, Vincent Arondel, Aruna Kilaru, Philip D. Bates, Nicholas A. Thrower, Christoph Benning, John B. Ohlrogge

**Affiliations:** 1 Department of Plant Biology, Michigan State University, East Lansing, Michigan, United States of America; 2 Great Lakes Bioenergy Research Center, Michigan State University, East Lansing, Michigan, United States of America; 3 Department of Biochemistry and Molecular Biology, Michigan State University, East Lansing, Michigan, United States of America; 4 Laboratoire de Biogenèse Membranaire, Université Bordeaux Segalen, Bordeaux, France; 5 Institute of Biological Chemistry, Washington State University, Pullman, Washington, United States of America; Instituto de Biología Molecular y Celular de Plantas, Spain

## Abstract

WRINKLED1 (*At*WRI1) is a key transcription factor in the regulation of plant oil synthesis in seed and non-seed tissues. The structural features of WRI1 important for its function are not well understood. Comparison of WRI1 orthologs across many diverse plant species revealed a conserved 9 bp exon encoding the amino acids “VYL”. Site-directed mutagenesis of amino acids within the ‘VYL’ exon of *At*WRI1 failed to restore the full oil content of *wri1-1* seeds, providing direct evidence for an essential role of this small exon in *At*WRI1 function. *Arabidopsis* WRI1 is predicted to have three alternative splice forms. To understand expression of these splice forms we performed RNASeq of *Arabidopsis* developing seeds and queried other EST and RNASeq databases from several tissues and plant species. In all cases, only one splice form was detected and VYL was observed in transcripts of all WRI1 orthologs investigated. We also characterized a phylogenetically distant WRI1 ortholog (*Eg*WRI1) as an example of a non-seed isoform that is highly expressed in the mesocarp tissue of oil palm. The C-terminal region of *Eg*WRI1 is over 90 amino acids shorter than *At*WRI1 and has surprisingly low sequence conservation. Nevertheless, the *Eg*WRI1 protein can restore multiple phenotypes of the *Arabidopsis* *wri1-1* loss-of-function mutant, including reduced seed oil, the “wrinkled” seed coat, reduced seed germination, and impaired seedling establishment. Taken together, this study provides an example of combining phylogenetic analysis with mutagenesis, deep-sequencing technology and computational analysis to examine key elements of the structure and function of the WRI1 plant transcription factor.

## Introduction

Many plant species accumulate triacylglycerol (TAG) in their seeds as a major storage component that provides carbon and energy for seedling development. These oils are also a staple in the human diet and are increasingly important as renewable feed stocks for industry. Currently, a wealth of information supports the pivotal role of WRINKLED1 (WRI1) in the regulation of plant seed oil biosynthesis. Seeds of the *Arabidopsis* *AtWRI1* (At3g54320) loss-of-function mutant *wri1-1* display an 80% reduction in TAG accumulation compared to wild-type (WT) [1]. WRI1 has been identified as a member of the APETALA2 (AP2) family of transcription factors [2,3]. Comparison of the transcriptome between developing WT and *wri1-1* seeds reveals that the majority of the genes which are expressed at reduced level in the *wri1-1* mutant are fatty acid and glycolytic enzymes [4]. Recent studies have confirmed a number of genes encoding enzymes involved in fatty acids synthesis and late glycolysis that are *WRI1* targets [5,6] which is also evidenced by high transcript co-expression for these genes with *WRI1* in *Arabidopsis* developing seeds [7] and *Zea mays* (*Z. mays*) [8]. . Specific motifs in the fatty acids synthesis genes to which the WRI1 protein binds have been characterized [5,9]. *WRI1* orthologs from *Z. mays* and *Brassica napus* (*B. napus*) have also been shown to function in plant oil biosynthesis [10,11,12]. Expression of *AtWRI1* and two *WRI1* orthologs identified from *Z. mays* are also able to restore the phenotypes of *wri1-1* and *wri1-4*, such as reduced seed oil content [2,11]. An increase in oil accumulation in transgenic *Arabidopsis* , *B. napus* or *Z. mays* seeds expressing *AtWRI1* or other *WRI1* orthologs has been reported [2,10,12]. In addition to its roles in regulating plant oil biosynthesis, *AtWRI1* is required for optimal seed germination and seedling establishment [13].

*AtWRI1* and *WRI1* orthologs from other plant species are highly expressed in seed tissue and coordinately expressed with fatty acid biosynthetic enzymes [2,10,11,14]. Recently, an ortholog of *WRI1* was found to be highly expressed in the mesocarp of oil palm (*EgWRI1*) [15,16]. The expressed sequence tag (EST) levels for *EgWRI1* are 50 fold higher than in date palm (which does not accumulate oil) and increase during fruit ripening in close coordination with increases in oil accumulation [15]. These data and the recent characterization of *At*WRI2, 3 and 4 [17] provide a strong indication that WRI1 and its homologs play similar roles in regulating fatty acid synthesis in both seed and non-seed tissues. *AtWRI3* and *AtWRI4*, that are expressed most highly in non-seed tissues, were shown to rescue the wrinkled seed and low oil phenotypes of the *wri1-3* mutant [17]. Although *EgWRI1* is highly expressed in fruit mesocarp, BlastP analysis of *Eg*WRI1 protein versus the *Arabidopsis* proteome indicates its highest similarity is with *At*WRI1, rather than *At*WRI2, 3, or 4.

Oil palm is a monocotyledonous plant that diverged from *Arabidopsis* >120 million years ago. Interestingly, the sequence of *Eg*WRI1 is 93 amino acids shorter than *At*WRI1 and exhibits low sequence identity over the C-terminal half of the proteins. These differences offered an opportunity to determine whether this divergent non-seed expressed *EgWRI1* is a functional *WRI1-*ortholog able to control oil synthesis and other phenotypes in a heterologous system.

Alternative splicing is an important form of regulation for many genes and contributes to the diversity of the transcriptome and proteome and thereby extends the range of functions exerted by single genes. Based on *in silico* analysis, approximately 6000 genes in *Arabidopsis* are predicted to have alternative splice forms, including approximately 340 transcription factor-encoding genes including *WRI1* [18]. The application of high-throughput transcript sequencing has provided direct evidence that at least 42% (~10,000) of *Arabidopsis* genes are alternatively spliced [19], 

Alternative splicing can occur in spatially and developmentally specific patterns, which are sometimes regulated through sensing environmental cues or stresses [20]. Some *Arabidopsis* genes express different splice variants in different tissues, in response to stress, and can also lead to differential subcellular localization of protein products [20,21]. Some *Arabidopsis* transcription factors repress their own function through producing alternative splice variants and by forming heterodimers [18,22]. Currently, three alternative splice forms are predicted for *AtWRI1* and it is unknown whether all three *AtWRI1* splice variants can be found in plant cells; or if present, whether these may have different patterns of expression or roles in plant fatty acid biosynthesis. The recent availability of very comprehensive EST and transcriptomic datasets for many *Arabidopsis* tissues provided the opportunity to evaluate the possible occurrence of the predicted alternative *AtWRI1* splice forms.

Here we show that expression of *EgWRI1* in the *Arabidopsis* *wri1-1* mutant is able to complement reduced seed oil content and other *wri1-1* impaired phenotypes. In addition, we provide evidence that *WRI1* splice form 3 (*At3g54320*.3) is the only form present in multiple *Arabidopsis* tissues. Furthermore, analysis of transcriptomic data from other species leads to a similar conclusion. The role of a small conserved exon of splice form 3 is further investigated.

## Results

### Expression of *HA-EgWRI1* rescues the low oil content in *wri1-1* seeds

**Figure 1:**
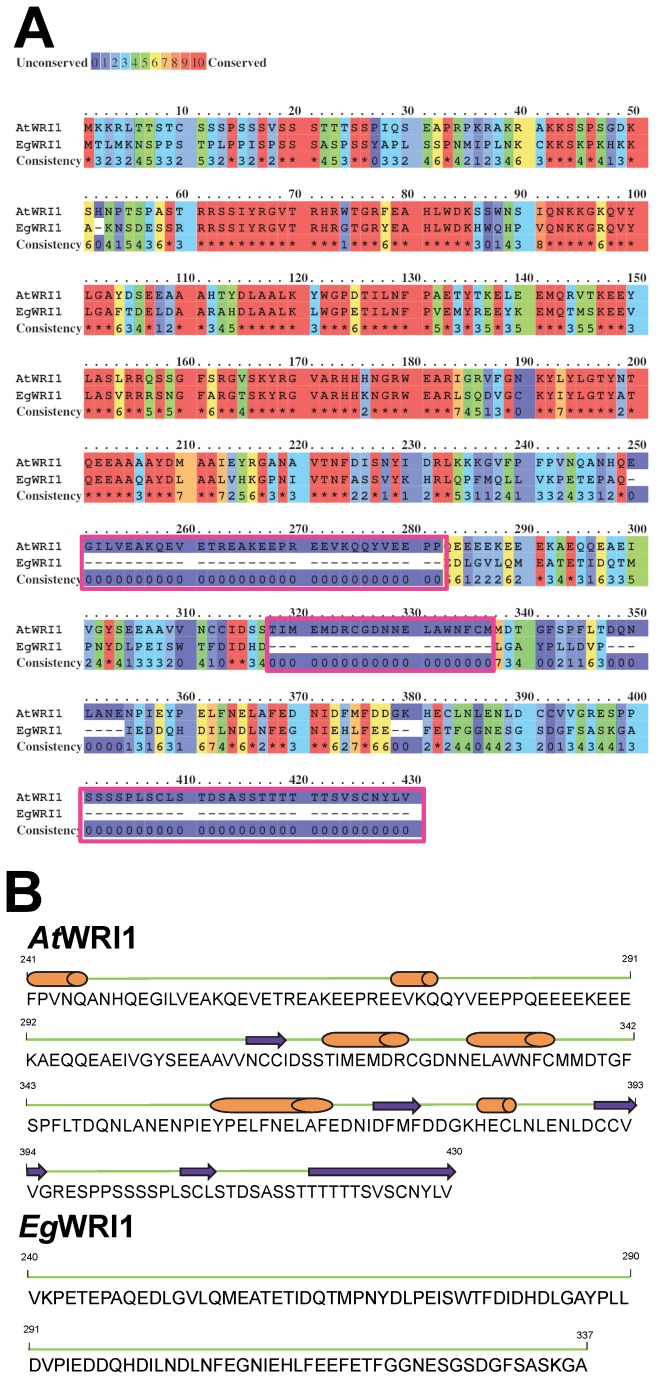
Alignment of protein sequence of *Eg*WRI1 and *At*WRI1. A) *Eg*WRI1 is 93 amino acids shorter compared to *At*WRI1. Most of the difference between protein sequences of *Eg*WRI1 and *At*WRI1 occurs at the C-terminal half of the protein (highlighted by boxes). Conservation of amino acids is denoted by different colors as illustrated by the scale bar. The alignment was analyzed by the PRALINE program (http://www.ibi.vu.nl/programs/pralinewww/). B) Secondary structure of *At*WRI1 and *Eg*WRI1 was analyzed by SWISS-MODEL (http://swissmodel.expasy.org/) and structure figure is manually generated based on the prediction. The orange cylinders and purple arrows indicate helix and extended-beta strands, respectively. Numerous helix and extended-beta structures that are found in the C-terminal of *At*WRI1 are missing in the C-terminal of *Eg*WRI1.

A comparison of protein sequences between *At*WRI1 and *Eg*WRI1 indicates that *Eg*WRI1 is 93 amino acids shorter compared to *At*WRI1 (Figure 1A). Almost all regions of high similarity between *At*WRI1 and *Eg*WRI1 are located in the N-terminal ~ 230 amino acids (Figure 1A). The C-terminal regions of *At*WRI1 (190 amino acids) and *Eg*WRI1 (98 amino acids) are strikingly diverged, (only 28% identical) with several large “deletions” within the *Eg*WRI1 sequence. Furthermore, the predicted secondary structures bear little resemblance; *At*WRI1 includes six helix regions in the C-terminal end of the protein, whereas *Eg*WRI1 possesses no predicted helix regions in the C-terminal region (Figure 1B).

To better understand features of the WRI1 structure that are important for WRI1 function we tested the ability of the divergent *EgWRI1* to restore seed oil and other phenotypes in the *Arabidopsis* *wri1-1* mutant background. Beyond *EgWRI1’s* ability to restore known *wri1-1* phenotypes, we also asked whether its expression might lead to additional phenotypes, such as higher oil accumulation. Such a result might occur if a heterologous protein ‘escapes’ repression that possibly acts on the C-terminus of the native protein or its mRNA.

**Figure 2:**
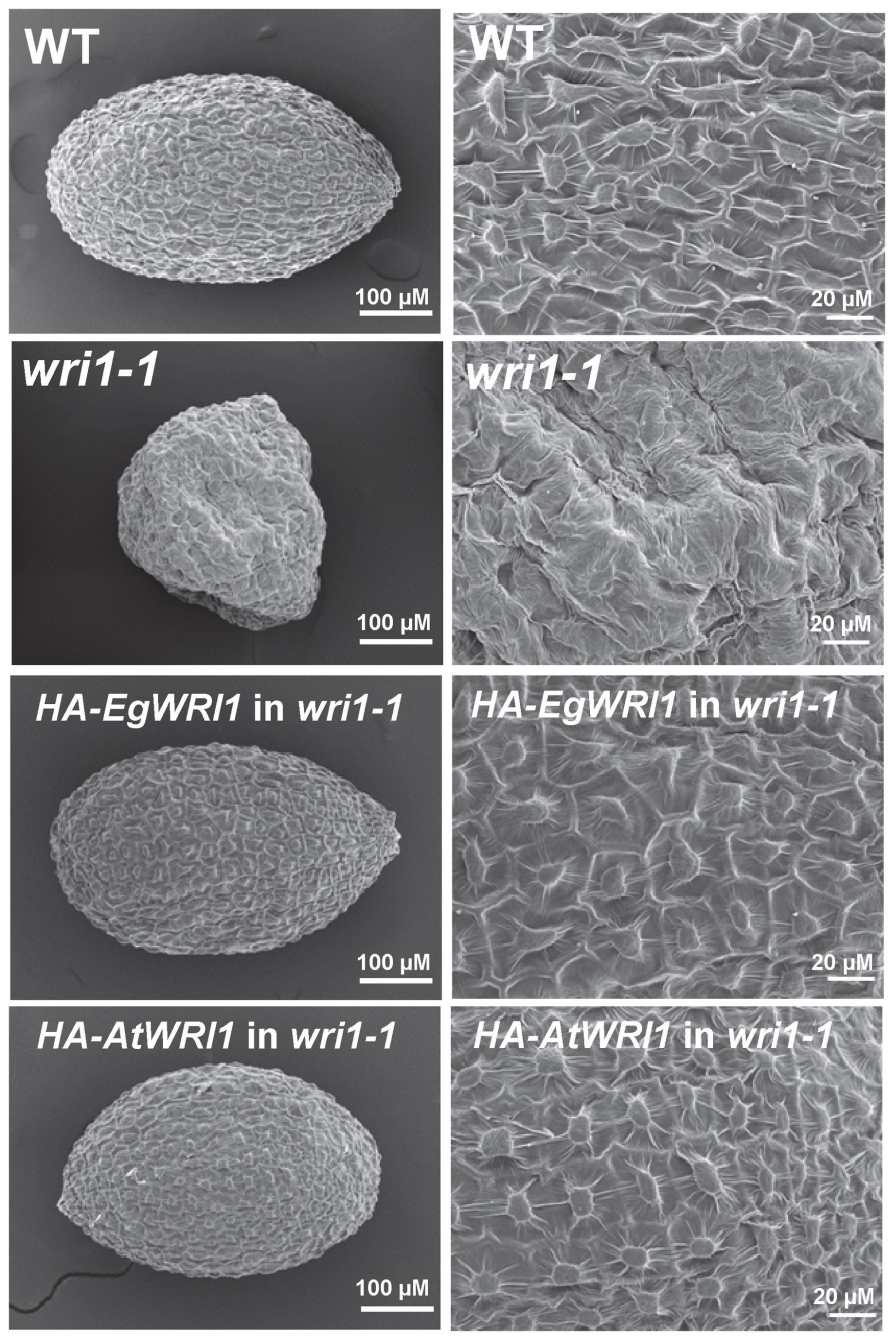
*HA-EgWRI1* (or *HA-AtWRI1*) complements the “wrinkled” feature of *wri1-1* seeds.

**Figure 3:**
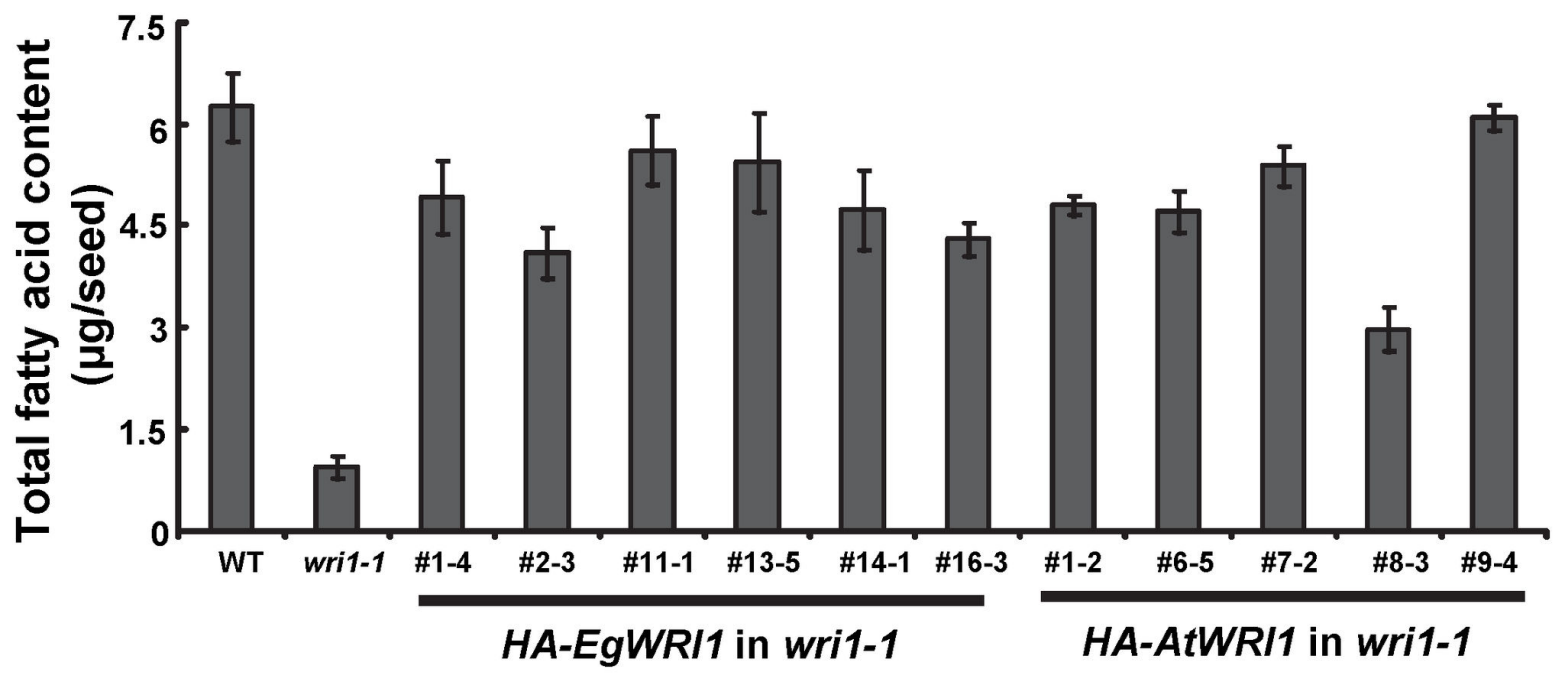
*HA-EgWRI1* rescues the reduced oil phenotype of *wri1-1* seeds. Total fatty acids in seeds of WT, *wri1-1* and transgenic *wri1-1* expressing *HA-EgWRI1* or *HA-AtWRI1* are shown in the figure. Results are shown as means ± SE (n = 3-4) of biological replications.

In order to investigate the ability of *EgWRI1* to restore several phenotypes observed in *wri1-1*, we generated transgenic plants expressing *HA-EgWRI1* under the constitutive cauliflower mosaic virus (CaMV) 35S promoter. As a positive control, experiments were conducted in parallel with transgenic *wri1-1* expressing *HA-AtWRI1*. As shown in Figure 2, homozygous transgenic *wri1-1* expressing *HA-EgWRI1* displayed a ‘non-wrinkled’ seed surface and normal seed shape indicating rescue of these phenotypes (also observed in transgenic *wri1-1* expressing *HA-AtWRI1*). We further measured the fatty acid content in seeds of *wri1-1* expressing *HA-EgWRI1*. As shown in Figure 3, expression of *HA-EgWRI1* restored the fatty acid content of *wri1-1* seeds, similar to *wri1-1* seeds rescued by expression of *HA-AtWRI1*. The fatty acid content in seeds of *HA-EgWRI1* transgenic lines (#1-4, #11-1, #13-5 and #14-1) compared to WT were not different with statistical significance (P > 0.05, t-test). In addition to its lower oil content, the fatty acid composition of *wri1-1* differs from WT in its lower relative content of 18:1 and higher content of 22:1 [1]. When the *wri1-1* mutant is complemented with *HA-AtWRI1* the fatty acid composition returns to a WT profile (Figure S1). In contrast, the fatty acid profile of *wri1-1* expressing *HA-EgWRI1* was only partially restored. Although, most fatty acids were not significantly different, the content of 18:1 and 22:1 were more similar to *wri1-1* than to WT (Figure S1). A similar result was observed in the complementation of *wri1* by *ZmWRI1* orthologs [11].

### Seed germination and impaired seedling phenotypes of *wri1-1* are restored by *HA-EgWRI1*

**Figure 4:**
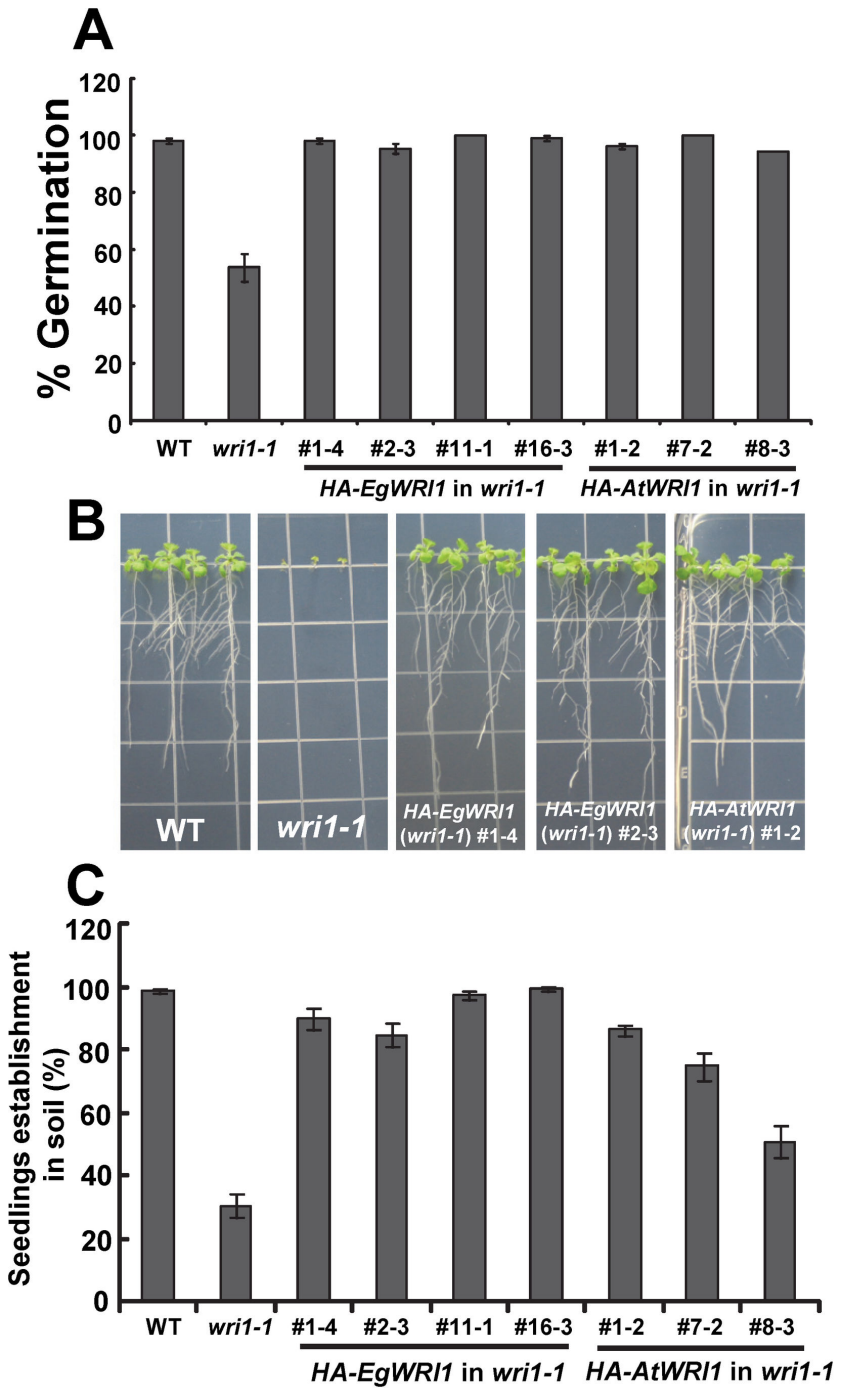
Seed germination and seedling establishment of *wri1-1* transformed with *HA-AtWRI1* or *HA-EgWRI1*. A) Expression of *HA-EgWRI1* rescues the reduced germination of *wri1-1* seeds. Results are shown as means ± SE (n = 3). Seed germination of all *HA-EgWRI1* transgenic lines were not significantly different compared to WT (P > 0.05, t-test). B) Expression of *HA-AtWRI1* or *HA-EgWRI1* in *wri1-1* complements the failure of seedling establishment in growth medium without sucrose. C) *HA-EgWRI1* rescues impaired seedling establishment of *wri1-1* plants in soil. Results are shown as means ± SE (n = 4). Seedling establishment of *HA-EgWRI1* transgenic lines (#1-4, #11-1, #16-3) did not differ significantly compared to WT (P > 0.05, t-test).

The *wri1-1* mutant exhibits reduced seed germination and impaired seedling establishment when germinated on medium without the addition of sucrose [13]. Expression of *HA-EgWRI1* corrected the reduced seed germination of *wri1-1* (Figure 4A). Transgenic *wri1-1* mutants expressing *HA-EgWRI1* established seedlings normally on agar-solidified medium lacking sucrose (Figure 4B) and on soil (Figure 4C).

As a further test of the influence of *WRI1* structural variants we also generated transgenic *wri1-1* expressing *EgWRI1-TAP* or *AtWRI1-TAP*. The low fatty acid content (Figure S2) and altered fatty acid profile of *wri1-1* seeds (Figure S3), reduced seed germination (Figure S5A) and impaired seedling establishment (Figure S4B) were all restored by *AtWRI1-TAP*. These results indicate that the fusion of the TAP tag at the C-terminus of *At*WRI1 did not cause any interference with the *At*WRI1 functions. In contrast, although the reduced seed germination of *wri1-1* was restored by *EgWRI1-TAP* (Figure S4A), this construct only partially restored the fatty acid content (Figure S2) and profile (Figure S3) of *wri1-1*. In addition, seedling establishment remained unsuccessful in transgenic *wri1-1* plants expressing *EgWRI1-TAP* (Figure S4B).

### Small exon “VYL” is essential for function of *At*WRI1

**Figure 5:**
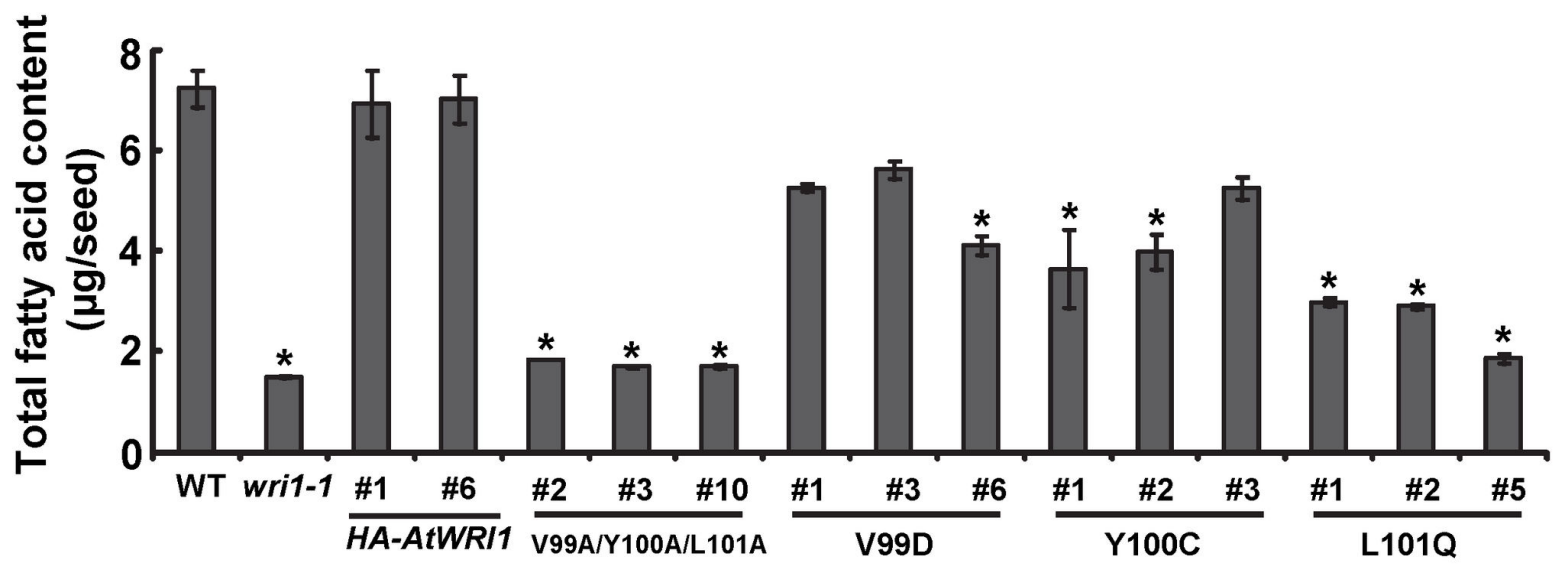
Fatty acid content of seeds of WT, *wri1-1* and transgenic *wri1-1* expressing *HA-AtWRI1* or *HA-AtWRI1s* mutated in VYL sequence. Results are means ± SE (n = 3). “*” indicates significant difference (P<0.05, t-test) between transgenic *wri1-1* lines expressing *HA-AtWRI1* (lines #1 and #6), and lines expressing the mutated genes.

The results above indicated that *Eg*WRI1 is able to complement the phenotypes of *wri1-1* mutant despite major differences in the C-terminal amino acid sequences of WRI1 from *Arabidopsis* and oil palm. A detailed analysis of features conserved between *At*WRI1, *Eg*WRI1 and other WRI1 orthologs from diverse species revealed a short protein sequence “VYL” (Figure S5A) present in the first AP2 domain (responsible for DNA-binding [23,24]). To better understand the function of the VYL sequence we asked if amino acid changes of "VYL" would lead to an impairment of *At*WRI1 function. A total of four versions of *At*WRI1 with amino acids substitutions (*At*WRI1^V99A/Y100A/L101A^; *At*WRI1^V99D^; *At*WRI1^Y100C^; *At*WRI1^L101Q^) were generated and used to transform the *wri1-1* mutant. Measurement of fatty acid content in these transgenic *wri1-1* seeds indicated that *At*WRI1^V99A/Y100A/L101A^ failed to restore the fatty acid content of *wri1-1*. The mutants *At*WRI1^Y100C^, or *At*WRI1^L101Q^, with single changes in the VYL sequence could partially, but not completely complement *wri1-1* fatty acid content (Figure 5). Taken together, these results indicate that mutation of residues encoded by this small exon lead to impairment of *At*WRI1 function and that “VYL” is an essential component of the *At*WRI1 structure.

### Analysis of *WRI1* alternative splice forms

**Figure 6:**
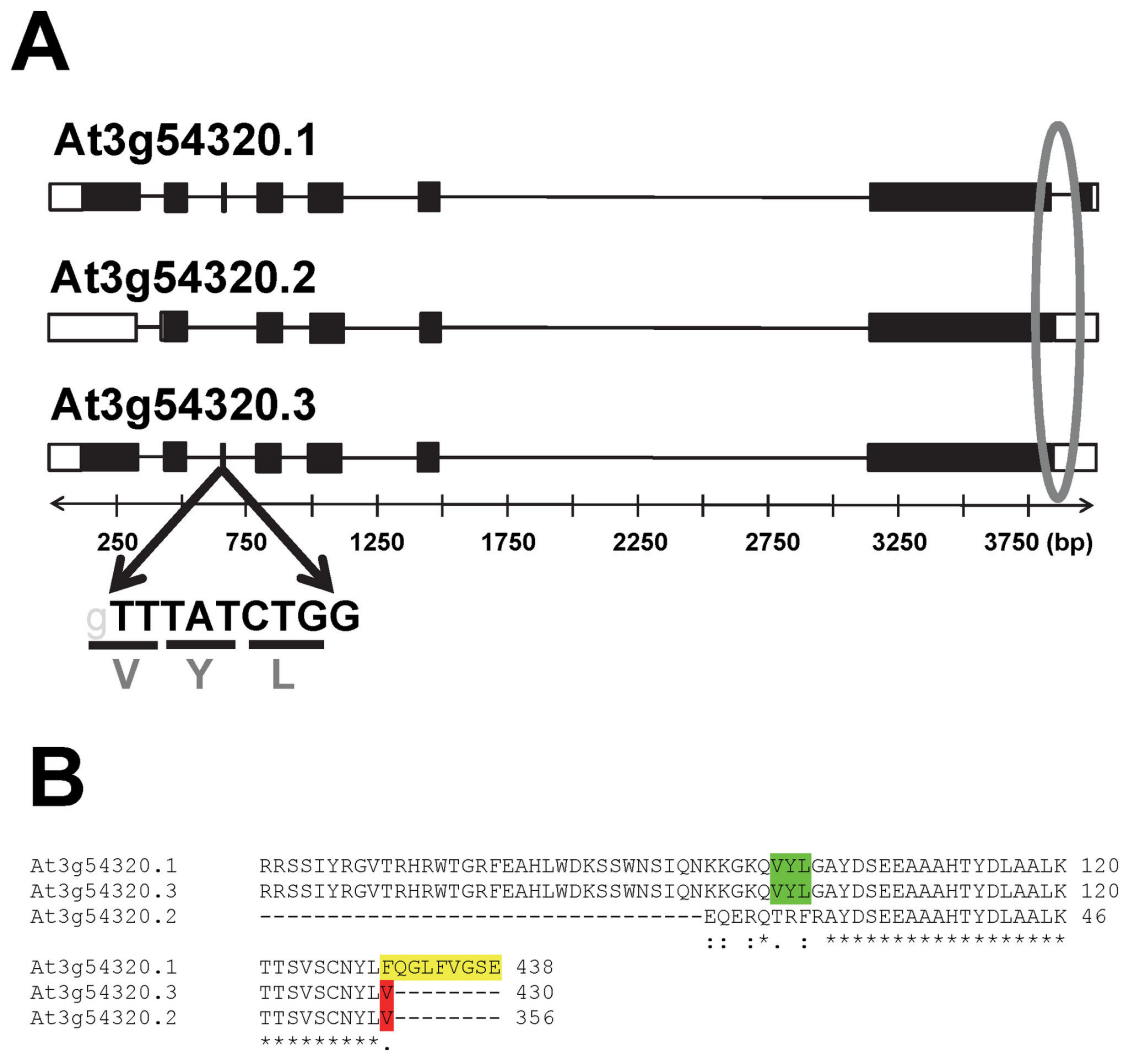
Three predicted alternative splice forms of *AtWRI1* (At3g54320.1, At3g54320.2, and At3g54320.3). A) Model of *AtWRI1* splice forms 1, 2 and 3. The notable features of these three forms are highlighted: (1) presence in splice forms 1 and 3 of a short 9 bp exon (TTTATCTGG) that encodes amino acids “VYL”; (2) presence of an intron at the 3’ end in splice form 1 and its absence in splice forms 2 and 3 is circled. B) Alignment of predicted protein sequence of three *AtWRI1* alternative splice forms. Exon 3 (amino acids sequence “VYL”) in *AtWRI1* splice forms 1 and 3 and absence from splice form 2 are highlighted in green. Unique amino acids “FQGLFVGSE” (form 1) and “V” (form 2 and 3) at the C-terminus of *At*WRI1 splice forms are highlighted in yellow and red, respectively.

In *At*WRI1, “VYL” is encoded by a 9 bp exon (Figure 6). Micro-exons of 2–25 bp are known to sometimes facilitate alternative splicing events [25]. *AtWRI1* is predicted to have three alternative splice forms (Figure 6) of different lengths and different protein sequences. Notably, “VYL” is absent from one of the predicted splice forms (At3g54320.2) In addition, Multiple Sequence Alignment examination of predicted protein sequences from approximately 34 plant genomes in the Phytozome database 9.0 (http://www.phytozome.org) indicated that VYL is also missing from the predicted *At*WRI1-orthologous amino acid sequences of 13 species (Figure S5B). These results indicate that the prediction of alternative protein sequences (or splice forms) for WRI1-like proteins occurs throughout the plant kingdom, and is not peculiar to *Arabidopsis* or its close relatives. We also note that 2 of 34 predicted amino acid sequences of WRI1 orthologs at Phytozome include “IYL” in place of “VYL”.

The three predicted alternative splice forms of *AtWRI1* (At3g54320.1, At3g54320.2 and At3g54320.3; http://www.arabidopsis.org) are referred to as splice form 1, 2 and 3 in this work. As highlighted in Figure 6, the distinguishing features of these three forms are: A) there is a short 9 bp exon near the N-terminus that encodes the amino acid sequence VYL. This exon is present in forms 1 and 3, but absent in form 2. B) In form 1, but not 2 or 3 there is an additional intron at the 3’ end of the coding sequence (circled). If removed by splicing, the stop codon is altered resulting in an additional 9 amino acids (FQGLFVGSE) at the C-terminus of form 1. C) Finally, in form 2, the first exon begins with a downstream ATG resulting in a protein that is 74 amino acids shorter. In the case of *AtWRI1*, the three splice forms are based on predicted gene models, rather than experimental evidence, but each has been previously assigned the same confidence level.

These predictions for *Arabidopsis* and for at least 33 other plant species with sequenced genomes, raised the question whether different *WRI1* splice forms are present in vivo and have different functions. We specifically asked: 1) Are all three *AtWRI1* splice forms expressed in *Arabidopsis* ? 2) Can we find multiple *WRI1* splice forms in other plant species? 3) If we can find multiple *WRI1* splice forms, are these expressed differently in different tissues? To evaluate the possible expression of multiple spice forms of *WRI1*, we tested directly by reverse transcription polymerase chain reaction (RT-PCR), performed RNASeq analysis of developing *Arabidopsis* seeds, and also searched publicly available large transcript databases.

### *AtWRI1* splice form 3 is the major form expressed in *Arabidopsis* seedlings

In many cases the presence of alternative splice forms can be detected by the different sizes of RT-PCR products [18,22]. We therefore performed RT-PCR on RNA extracted from young *Arabidopsis* seedlings (3-to-9-day-old) and with primers targeted at the short 9 bp exon and the last 3’ intron. Because expression of *AtWRI1* is found to be sugar-inducible [3], we grew *Arabidopsis* on medium with or without the addition of sucrose, to determine if different *AtWRI1* splice forms might be expressed differently under these two conditions. A primer set (FW1 + RV1) was designed to amplify a PCR product of 157bp (in splice form 1 or 3) or 148 bp (in splice form 2) respectively (Figure S6A). Sequencing of fourteen independent PCR products (Figure S6B) indicated in all cases, the presence of *AtWRI1* exon 3.

The primer set (FW2 + RV2) was designed to amplify a PCR product of 269 bp (in form 2 or 3) or 167 bp (in form 1) respectively. As shown in Figure S6C, all the samples that we tested supported the existence of form 2 or 3 (a PCR product of 269bp). A PCR product of form 1 (167bp) was not detected. Further sequencing of each PCR product in Figure S6C also confirmed this conclusion. Combining the results, this evidence indicated that splice form 3 of *AtWRI1* is the major form expressed in *Arabidopsis* seedlings.

As a second approach to evaluate expression of alternative splice forms in *Arabidopsis* , we searched a number of databases of Sanger ESTs, 454 pyrosequencing and Illumina RNASeq. Although large EST and RNASeq databases are available from several *Arabidopsis* tissues, the datasets do not include large numbers of reads from developing seeds, where *WRI*1 is most highly expressed. Therefore, we performed RNASeq analysis on three stages of developing seeds. Approximately 100 million 50 nt Illumina reads were mapped to the TAIR10 genome and analyzed. From these data, *WRI1* was expressed at a level of 165 Fragments per Kilobase per Million fragments (FPKM) (average of three developmental stages). This represents approximately 0.01% of the mRNA population and provides a more quantitative estimate of *WRI1* expression than previously available for *Arabidopsis* developing seeds. Over 500 WRI1 reads mapped to the region of the *WRI1* gene that overlaps the 9 bp exon 3. A section of the reads is presented in GBrowse format (Figure S7). No reads were detected that lacked exon 3. We also examined the 3’ sequences and found no sequences for splice form 1. Based on the number of reads representing splice form 3 and no reads representing forms 1 or 2, a binomial test indicated high probability (P > 0.999) that splice forms 1 or 2 of *WRI1* are not expressed in *Arabidopsis* developing seeds.

Alternative splice forms of genes are sometimes expressed only in specific tissues [20,21]. To evaluate this possibility, we also examined publicly available Ilumina RNASeq data from *Arabidopsis* roots and flowers. Although *WRI1* is expressed at much lower levels in these tissues (13.1 FPKM root, 10.9 FPKM flower), the 9 bp exon 3 was represented by over 80 RNASeq reads from roots and 20 reads from flowers. No RNASeq reads were found that lacked exon 3. Examination of the 3’ end (represented by 130 RNASeq reads in roots and 60 reads in flowers) revealed that the predicted 3’ intron was not spliced out in any reads. Thus, splice form 1 was also not detected in either root or flower datasets. Taken together, although expression of the alternative splice forms at very low levels cannot be ruled out, these data indicate that splice forms 1 or 2 are unlikely to play a biological role in *Arabidopsis* flowers and roots grown under standard condition.

### Analysis of *WRI1* alternative splice form expression in plant species other than *Arabidopsis*

The presence/absence of the 9 bp short exon in *WRI1* was also examined for a number of other plant species. We chose a 69 nt sequence which includes exon 3 with 60 additional bp of flanking sequence (Figure S8A) to search 2.2 million pyrosequencing (454) ESTs from developing seeds of *B. napus* [14]. More than 200 of these ESTs were identified as *WRI1* orthologs and of these > 80 spanned the exon 3 region. All of these reads included sequences that represent exon 3. Similarly, in the analysis of > 0.9 million castor ESTs [14], > 50 of these ESTs spanned the 5’ sequence region and all contained the VYL sequence. Finally, from analysis of > 4 million ESTs of oil palm mesocarp [15], > 200 ESTs spanned the exon 3 region, and all encoded ‘VYL”. 

In addition, an analogous procedure was used to identify splicing at the 3’-end that distinguishes splice form 1 from splice forms 2 & 3. A search sequence of 32 nt located just before the last intron (spliced out in *AtWRI1* splice form 1; Figure S9A), was used to identify *B. napus* ESTs that encoded the 3’ end (see Figure S9B). In the *B. napus* EST database, 80 ESTs were identified that closely matched the 3’ sequence of *WRI1* and in all cases the stop codon position matched that of *AtWRI1* splice forms 2 and 3, but not 1. The same conclusion was reached by analysis of 90 castor ESTs containing the 3’ stop codon. Taken together, for *B. napus*, castor and oil palm, we could not detect evidence for splice forms other than those corresponding to *WRI1* splice form 3.

## Discussion

### Monocot non-seed *EgWRI1* is functional in dicot *Arabidopsis* plant

In addition to *AtWRI1*, the seed-expressed *BnWRI1* or *ZmWRI1* orthologs have been confirmed to function in regulation of seed oil biosynthesis by their ability to complement *wri1* or to increase seed oil content [10,11,12]. Recently two *AtWRI1* homologs (*AtWRI3* and *AtWRI4*) that are stem and flower expressed, were shown to also activate expression of fatty acid biosynthetic genes and to complement the *wri1* mutant [17]. In this study we asked whether a more highly diverged WRI1 ortholog associated with very high oil levels in non-seed tissue might have evolved different properties associated with this function. It was also unknown whether this shorter and divergent monocot non-seed *EgWRI1* could function in regulating seed oil synthesis in a dicot or result in different phenotypes in germination and seedling development. Whether *ZmWRI1a* and *ZmWRI1b* can complement germination and seedling development phenotypes of *wri1* has not been reported. Our work indicated that expression of *EgWRI1* is able to rescue the ‘wrinkled’ seed coat (Figure 2), reduced seed oil content (Figure 3) and other phenotypes of *wri1-1* (Figure 4). Interestingly, we noticed that the seed fatty profile of transgenic *wri1-1* expressing *EgWRI1* is not completely rescued compared to *wri1-1* expressing *AtWRI1* (Figure S1). Previous work also found that expression of *ZmWRI1a* or *ZmWRI1b* in a *wri1* mutant did not restore the fatty acid profile to that of WT [11]. However, other than the phenotype of fatty acid profiles in transgenic *wri1* plants, there were no differences found between transgenic *wri1-1* plants expressing *EgWRI1* or *AtWRI1*, in restoring *wri1* mutant phenotypes to WT (Figures 2-4). These results indicate that despite major differences in their primary and secondary structure, this diverged *EgWRI1* functions similarly to *AtWRI1* in the *wri1-1* mutant.

A question that is raised by this study concerns the function of the C-terminal regions of *Eg*WRI1 and other WRI1 proteins, which are likely activation domains interacting with other factors of the transcription complex [26]. The highest sequence similarity between *EgWRI1* and *At*WRI1 is located in the N-terminus (~ 230 amino acids), which includes the crucial AP2 domains, a highly conserved feature in all AP2-type transcription factors. *Eg*WRI1 is 93 amino acids shorter than *At*WRI1 (Figure 1) and 92 of the amino acid ‘deletions’ are in the C-terminal region. After the AP2 domains, *Eg*WRI1 is 111 amino acids long, compared to 203 amino acids for *At*WRI1. In this study, we observed that fusion of an approximately 20 kDa protein tag (TAP) at the C-terminal region of *Eg*WRI1 resulted in only minor restoration of the *wri1-1* seed oil and wrinkled phenotypes (Figure S2-S4). However, *Eg*WRI1-TAP retained some function, based on the fact that reduced germination of *wri1* was still rescued (Figure S4A). In contrast, *At*WRI1-TAP successfully complemented all phenotypes of *wri1-1* that we examined.

### Two predicted WRI1 splice forms are likely ‘artifacts’ of gene prediction software

Alternative splicing is an important form of regulation for many genes and can increase the functional diversity of transcripts in eukaryotic cells. Evidence presented in this manuscript indicates that splice form 3 of *WRI1* is the only splice form that can be found in developing seeds, roots, flowers, and young seedlings of *Arabidopsis* and in developing seeds of *B. napus* and castor. Alternative splice forms in some cases produce protein products that lack or add functional domains, expanding their ability to interact within diverse cellular processes. For example, a splice form of *Arabidopsis* *IDD14β* encodes a product lacking a functional DNA-binding domain but plays a role in attenuating the activity of the full-length *IDD14α* through the formation of heterodimers [18]. Similarly, *Arabidopsis* *CCA1* also produces a shortened alternative splice variant that results in the formation of nonfunctional heterodimers, decreased DNA binding activity and alters the circadian rhythm [22]. These and other examples, together with the alternative predicted forms of *WRI1* raised the intriguing possibility that plant cells might use a similar self-regulatory mechanism of repressing or otherwise modifying *WRI1* function through expressing alternative splice forms. However, all of our experimental evidence and bioinformatic searches failed to detect the presence of predicted *WRI1* splice form 2 or 3.

The presence of *WRI1* splice form 1 represented by one EST from suspension cultures treated with cycloheximide indicates that alternative splicing of *WRI1* can occur. However, since alternative splice variants can be produced due to splice errors induced by stress [20], it is likely that cycloheximide led to aberrant splicing that is reflected by this one EST. Given the fact that *WRI1* splice form 1 has been observed in only this one case and differs by only eight additional amino acids at the *WRI1* C-terminal region (Figure 6B), we consider that a specific function of *WRI1* splice form 1 under normal plant growth is very unlikely.

Taken together, we conclude that the *WRI1* splice form 1 and 2, while predicted by automated annotation at the same confidence level as splice form 3, are unlikely to play a role in gene regulation, in the diverse tissues we examined. The prediction of forms 1 and 2 may be related to the fact that the sequences at the intron/exon junctions are not ‘cannonical’ which can lead to incorrect exon identification [27,28]. This ‘error’ in gene prediction is not specific to *Arabidopsis* because in the Phytozome database approximately one third of predicted protein sequences of WRI1 orthologs lack the VYL sequence (Figure S5B).

### Small exon encoded “VYL” residues are a key component of AtWRI1 function

“VYL” is conserved in a number of WRI1 orthologs discovered in many plant species (Figure S5). The possible involvement of the “VYL” sequence of *At*WRI1 in alternative splicing of WRI1 was discussed by Masaki et al., (2005), but biological or functional evidence to demonstrate a role of VYL in plants was not provided. Krizek (2003) used yeast to test the ability of a randomly mutagenized population of the *Arabidopsis* AP2-type transcription factor, AINTEGUMENTA (ANT) to bind to an ANT target sequence in yeast. Mutation of either “Y” or “L” lead to a full impairment of transcription activation while mutation of “V” lead to a reduced activation of ANT [29]. In the present study, we show that several mutated forms of residues “VYL” lead to failure or impaired ability to complement the low seed oil content of *wri1-1* mutant (Figure 5) thus providing functional evidence in plants for the essential role of “VYL” for *At*WRI1 function.

"VYL" is not a unique feature of WRI1-like transcription factors. The alignment of *Arabidopsis* AP2-type transcription factors indicates that all 18 members of the AP2 transcription factor family have a conserved protein sequence of "VYL" (http://planttfdb.cbi.pku.edu.cn/msa.php?sp=At&fam=AP2). Notably, amino acid “L” is more highly conserved and presumably most critical in AP2-type transcription factors. The results shown in Figure 5 supports this hypothesis by demonstrating that expression or HA-*At*WRI1^L101Q^ in *wri1-1* was the least effective in rescue of *wri1-1* oil content, compared to HA-*At*WRI1^V99D^ or HA-*At*WRI1^Y100C^ (Figure 5).

In summary, this study has provided new insights into WRI1 structure at the protein and transcript level. The *Eg*WRI1 sequence identified from oil palm mesocarp is highly similar to *At*WRI1 over the N-terminal 230 amino acids, but surprisingly divergent in sequence, in length and in predicted secondary structure over the remainder of the protein’s C-terminal domain. Nevertheless, the *Eg*WRI1 protein is functional in restoring oil content, germination and seedling establishment of the *Arabidopsis* *wri1-1* mutant, implying that the C-terminus may have a more general role in maintaining WRI1 structure/function, rather than, for example, interactions with specific DNA sequences. Second, the conserved VYL small exon of WRI1 was shown to be essential for full WRI1 function. Mutations of all three residues caused complete loss of ability to complement *wri1-1*, and single mutations at the “L” residue were more negative than at V or Y. Finally, we have established that splice form At3g54320.3 is the only one of the three predicted splice forms that is expressed under normal growth conditions of seeds, roots, flowers and seedlings of *Arabidopsis* .

## Materials and Methods

### Plant Materials

*Arabidopsis* (*Arabidopsis thaliana*) wild-type (Columbia-2 ecotype) and *wri1-1* [1] were used in this study. Plants were grown in a growth chamber on potting mix at 22°C with a 16 h light (100-150 µmol m^−2^ s^−1^ illumination)/8 h dark photoperiod cycle. For experiments with plants grown on plates, seeds were surface sterilized in 70% (v/v) ethanol (containing 0.05% (v/v) Tween 20), following by rinsing in 95% (v/v) and pure ethanol. Sterilized seeds were spread on petri dishes containing half-strength Murashige and Skoog (MS) medium (Caisson), 2.6 mm MES (pH 5.7; adjusted with KOH), 1% sucrose (unless noted otherwise in the figure legends), and 0.8% agar. Seeds were stratified at 4°C in the darkness for 2-3 d prior to use.

### Plasmid Construction and *Arabidopsis* Transformation

The oil palm *EgWRI1* gene was synthesized by GeneArt based on cDNA sequences obtained from oil palm mesocarp [15]. The nucleotide sequence of synthetic *EgWRI1* is in Figure S10. Site-directed mutations (*At*WRI1^V99A/Y100A/L101A^; *At*WRI1^V99D^; *At*WRI1^Y100C^; *At*WRI1^L101Q^) were introduced into the *AtWRI1* coding sequence (CDS) by PCR (see Table S1). The modified genes were subcloned into binary vectors pEarleyGate 201 or pEarleyGate 205 [30]. Constructs were introduced into *wri1-1* mutants through *Agrobacterium tumefaciens* (GV3101 strain)-mediated transformation by floral dipping [31]. Transgenic seedlings were selected with 10 µg/mL Basta (Sigma-Aldrich) on plates. Genomic DNA of transgenic seedlings was extracted and gene insertion was confirmed by PCR using a 35S promoter forward primer and a gene-specific reverse primer. Homozygous plants were used in all experiments except T2 transgenic plants in experiments presented in Figure 5.

### Scanning electron microscopy

Sample preparation followed methods previously described [2]. Samples were examined in a JEOL 6610LV SEM (tungsten hairpin electron emitter) scanning electron microscope (JEOL Ltd.).

### Germination and Seedling Establishment Assays

Surface-sterilized seeds were spread on half-strength MS plates (containing 1% sucrose). Seeds were stratified for 2-3 d prior to being transferred to a growth chamber. Germination was scored by radicle emergence 2 d after imbibition. To determine seedling establishment, plants were grown vertically on half-strength MS plates (without the addition of sucrose) and 10-day-old seedlings were evaluated for seedling establishment. Alternatively, seeds were grown in potting mix and two-week-old seedlings were used to count the number of the seedlings that had established.

### RT-PCR for *WRI1* transcript amplification and analysis

Whole seedlings were harvested from plates, ground in liquid nitrogen, and total RNA was isolated using the RNeasy Plant Mini kits (Qiagen). Genomic DNA contamination was removed using DNase I (Qiagen). First-strand cDNA was synthesized using the Reverse Transcription System (Promega). Genomic DNA contamination was not found in the RNA samples treated with DNase I. PCR products were purified using the Gel and PCR Clean-Up System (Promega).

### RNASeq analysis of *WRI1* transcripts from *Arabidopsis*

Developing seeds were collected from liquid nitrogen frozen siliques at 7-8, 9-10, and 11-12 d after flowering. Siliques were opened over dry ice and frozen seeds were separated from the silique walls by filtering through a liquid nitrogen cooled sieve into a tube on dry ice. Approximately 100 mg of developing seeds was finely ground and RNA extracted as described [32] and analyzed for yield and quality by capillary electrophoresis (Agilent 2100). Libraries for sequencing were prepared from 2–4 µg total RNA using Illumina TruSeq RNA kits and sequenced with Illumina HiSeq2000. Reads (50 nt) were trimmed, filtered and aligned to TAIR10 using TopHat v 1.4.1 (Parameters: --no-novel-juncs; -G TAIR10.gff) and Bow tie v 0.12.7. Cufflinks v 2.0.2 was used to generate gene FPKM expression measures. The results were also loaded into a genome browser for inspection. Three different read mappings were performed to assess WRI1 alternative splice forms. Samples were aligned to TAIR10 using the CLC Genomics, Map Reads to Reference Tool and Large Gap Read Mapping Tool (Parameters: Mismatch cost 2; Insertion cost 3; Deletion cost 3; Similarity 0.9; Length fraction 0.9). 117 million RNASeq reads for roots (SRR331219,SRR331224) and 58.4 million for flowers (SRR388668, SRR388670, SRR013413, SRR013416) were downloaded from the NCBI short read archive and mapped to TAIR10 as described above.

### Fatty Acid Analysis

*Arabidopsis* seed oil content analysis followed the method described previously [33], with minor modification. In brief, twenty *Arabidopsis* seeds were transesterified directly in a glass tube by addition of 1 mL freshly prepared sulfuric acid in methanol (5% (v/v)), 25 µL of BHT solution (0.2% butylated hydroxy toluene in methanol), 25 µg of triheptadecanoin (as internal standard) and 300 µL of toluene. After reaction at 90°C for 90 min the fatty acid methyl ester extracts were extracted and analyzed by gas chromatography with a DB23 column.

### Statistical Analysis

A binomial test using R programming was used to calculate the statistical significance of conclusions on *AtWRI1* splice form abundance. The student’s t-test was performed to evaluate the statistical confidence in differences observed between controls and samples expressing different *WRI1* constructs.

## Supporting Information

Figure S1 Profiles of seed fatty acid composition of WT, *wri1-1* and *wri1-1* expressing *HA-EgWRI1* and *HA-AtWRI1*. Six independent transgenic lines overexpressing *HA-EgWRI1*(#1-4, #2-3, #11-1, #13-5, #14-1, and #16-3, respectively; from left to right) and five independent transgenic lines overexpressing *HA-AtWRI1* (#1-2, #6-5, #7-2, #8-3, and #9-4, respectively; from left to right) are shown above. Results are means ± SE (n = 3-4).
(PDF)

Figure S2 C-terminal TAP-tagged *AtWRI1* rescues the reduced oil phenotype of *wri1-1* mutant. However, C-terminal TAP-tagged *EgWRI1* fails to rescue the reduced oil of *wri1-1*. Results are means ± SE (n = 3-4). “*” indicates significant difference (P<0.05, t-test) between WT and other plants.
(PDF)

Figure S3 Profiles of seed fatty acid composition of WT, *wri1-1* and *wri1-1* overexpressing *EgWRI1-TAP* and *AtWRI1-TAP*. Four independent transgenic lines overexpressing *EgWRI1-TAP* (#2-6, #4-6, #6-2, and #7-1 respectively; from left to right) and four independent transgenic lines expressing *AtWRI1-TAP* (#1-3, #4-5, #6-5, and #7-2, respectively; from left to right) are shown below. Results are shown as means ± SE (n = 3-4).
(PDF)

Figure S4 Phenotypes of *wri1-1* plants expressing *EgWRI1-TAP* or *AtWRI1-TAP*. **A**) C-TAP-tagged *EgWRI1* and *AtWRI1* were both able to complement the reduced germination of *wri1-1* seeds. Results are shown as means ± SE (n =3-4). The seeds germination of *EgWRI1-TAP* transgenic lines compared to WT were not significantly different (P > 0.05, t-test). **B**) Transgenic *wri1-1* plants expressing *AtWRI1-TAP* or *EgWRI1-TAP*. Plants were grown in medium without the addition of sucrose.
(PDF)

Figure S5 Alignment of WRI1 orthologs. **A**) Alignment of section of AP2 domain of WRI1 orthologs from *B. napus*, maize and oil palm indicating the most conserved amino acids across different WRI1s. Amino acids “VYL” (highlighted by a red box), are highly conserved in plant WRI1s. **B**) Alignment of WRI1 ortholog amino acid sequences predicted from genome sequencing information at Phytozome (http://www.phytozome.org/). Of the 34 predicted WRI1-like protein sequences, amino acid sequence “VYL” (highlighted by red box) is missing in 13. The locus IDs of predicted WRI1 orthologs are as follows. *M.esculenta* (cassava4.1_029667m.g); *R.communis* (30069.t000002); *L.usitatissimum* (Lus10008939.g); *P.trichocarpa* (Potri.008G011900); *P.vulgaris* (Phvul.011G187400); *G.max* (Glyma08g24420); *C.sativus* (Cucsa.282940); *P.persica* (ppa023152m.g); *M.domestica* (MDP0000186581); *F.vesca* (gene00377-v1.0-hybrid); *A.thaliana* (AT3G543200; *A* *. lyrata* (485830); *C* *. rubella* (Carubv10018845m.g); *B* *. rapa* (Bra007066); *T. halophila* (Thhalv10010394m.g); *C. papaya* (evm. TU. supercontig_54.28); *G* *. raimondii* (Gorai.011G225700); *T* *. cacao* (Thecc1EG044588); *C. sinensis* (orange1.1g036423m.g); *C* *. clementina* (Ciclev10003896m.g); *E* *. grandis* (Eucgr.J00316); *V. vinifera* (GSVIVG01020066001); *S. tuberosum* (PGSC0003DMG400027502); *S* *. lycopersicum* (Solyc01g096860.1); *M* *. guttatus* (mgv1a007319m.g); *A* *. coerulea* (Aquca_016_00348); *S. bicolor* (Sb02g025080); *Z. mays* (GRMZM2G141219); *S* *. italica* (Si030129m.g); *P* *. virgatum* (Pavirv00024549m.g); *O. sativa* (LOC_Os11g03540); *B* *. distachyon* (Bradi4g30617); *S* *. moellendorffii* (85823); *P* *. patens* (Pp1s32_65V6).
(PDF)

Figure S6 Analysis of *AtWRI1* splice forms by RT-PCR. **A**) Two pairs of PCR primers were designed, which cover the region of *AtWRI1* exon3 and last intron, respectively. The length of PCR product with primers set 1 (FW1+RV1) is 157bp (splice form 1 and 3) and 148bp (splice form2), respectively. The length of PCR product with primer set 2 (FW2+RV2) is 167bp (splice form2; last intron is spliced out) and 269bp (splice form 2 and 3; last intron is not spliced out), respectively. *AtWRI1* transcript accumulation in *Arabidopsis* plants was analyzed by semi-quantitative RT-PCR, with primers set 1 (B) and primers set 2 (C), respectively. Samples with even numbers were grown in growth medium containing 3% sucrose. Samples with odd numbers were grown in growth medium without the addition of sucrose. *Arabidopsis* seedlings are 3- (sample 1 & 2), 4- (sample 3 & 4), 5- (sample 5 & 6), 6- (sample 7 & 8), 7- (sample 9 & 10), 8-(sample 11 & 12), and 9- (sample 13 & 14) day-old, respectively.
(PDF)

Figure S7 Alignment of Illumina RNASeq reads from mRNA of developing seeds of *Arabidopsis* . We analyzed >100 million Illumina reads from developing *Arabidopsis* seeds. Of these, ~10,000 or 1% mapped to the *AtWRI1* gene. 500 of these reads mapped to the genome region that included the 9 bp exon 3. A subset of the reads is presented based on visualization of alignment by GBrowse (http://www.gbrowse.org). No reads were detected that lacked exon 3. Similar analysis of 3’ sequences indicated only splice form 3 was represented.
(PDF)

Figure S8 Diagnostic search sequence for exon 3. **A**) A 69 nucleotide sequence was designed to distinguish *AtWRI1* splice form 1 and 3 from form 2. The sequence includes the nine nucleotides that encode “VYL” (highlighted in green), together with 30 nucleotides 5’ and 3’ flanking sequences. **B**) Position of diagnostic search sequence is highlighted by red boxes in the picture of alignment of predicted partial cDNAs of three *AtWRI1* alternative splice forms.
(PDF)

Figure S9 Diagnostic search sequence for *AtWRI1* 3’ end. **A**) The flanking sequence upstream from the last intron which is spliced out in *AtWRI1* splice form 1 was chosen as a diagnostic search sequence for the 3’ end splice form search. **B**) Position of the diagnostic search sequence for *WRI1* 3’ end is highlighted by red boxes in alignment of predicted partial cDNAs of three *AtWRI1* alternative splice forms.
(PDF)

Figure S10 Nucleotide sequence of synthetic *EgWRI1*. (PDF)

Table S1 Primers used in this study. (PDF)
