# Supplementary material for: WRINKLED1, A Ubiquitous Regulator in Oil Accumulating Tissues from Arabidopsis Embryos to Oil Palm Mesocarp
Source: PLoS One. 2013 Jul 26;8(7):e68887. doi: 10.1371/journal.pone.0068887 (PMC3724841; doi:10.1371/journal.pone.0068887)
Supplement: Figure S1 — Six independent transgenic lines overexpressing HA-EgWRI1(#1-4, #2-3, #11-1, #13-5, #14-1, and #16-3, respectively; from left to right) and five independent transgenic lines overexpressing HA-AtWRI1 (#1-2, #6-5, #7-2, #8-3, and #9-4, respectively; from left to right) are shown above. Results are means ± SE (n = 3-4). (PDF) [file pone.0068887.s001.pdf]

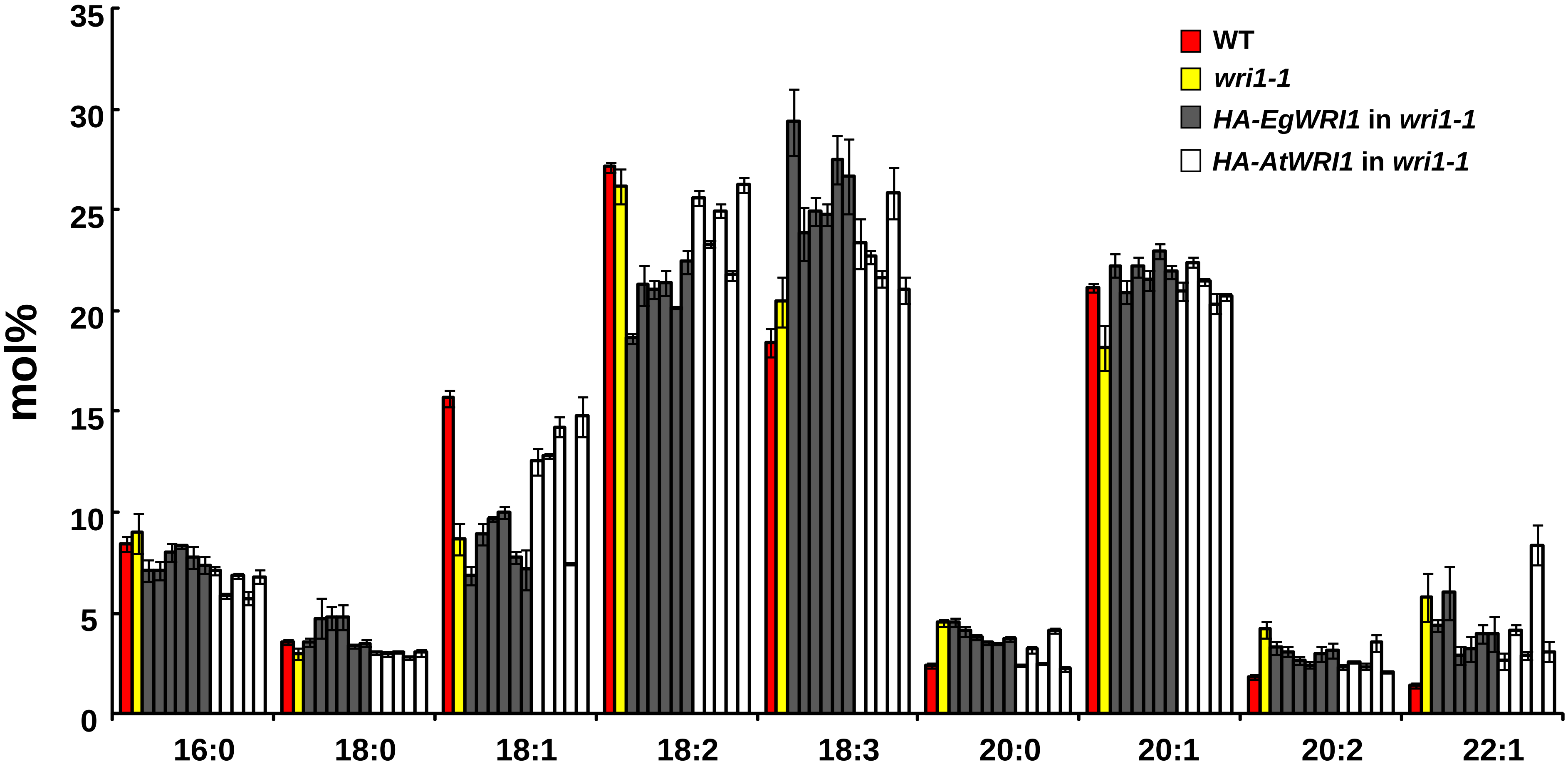

Figure S1. Profiles of seed fatty acid composition of WT, *wri1-1* and *wri1-1* expressing *HA-EgWRI1* and *HA-AtWRI1*. Six independent transgenic lines overexpressing *HA-EgWRI1* (#1-4, #2-3, #11-1, #13-5, #14-1, and #16-3, respectively; from left to right) and five independent transgenic lines overexpressing *HA-AtWRI1* (#1-2,#6-5,#7-2,#8-3, and #9-4, respectively; from left to right) are shown above. Results are means ± SE (n = 3-4).
