# Supplementary material for: WRINKLED1, A Ubiquitous Regulator in Oil Accumulating Tissues from Arabidopsis Embryos to Oil Palm Mesocarp
Source: PLoS One. 2013 Jul 26;8(7):e68887. doi: 10.1371/journal.pone.0068887 (PMC3724841; doi:10.1371/journal.pone.0068887)
Supplement: Figure S2 — However, C-terminal TAP-tagged EgWRI1 fails to rescue the reduced oil of wri1-1. Results are means ± SE (n = 3-4). “*” indicates significant difference (P<0.05, t-test) between WT and other plants. (PDF) [file pone.0068887.s002.pdf]

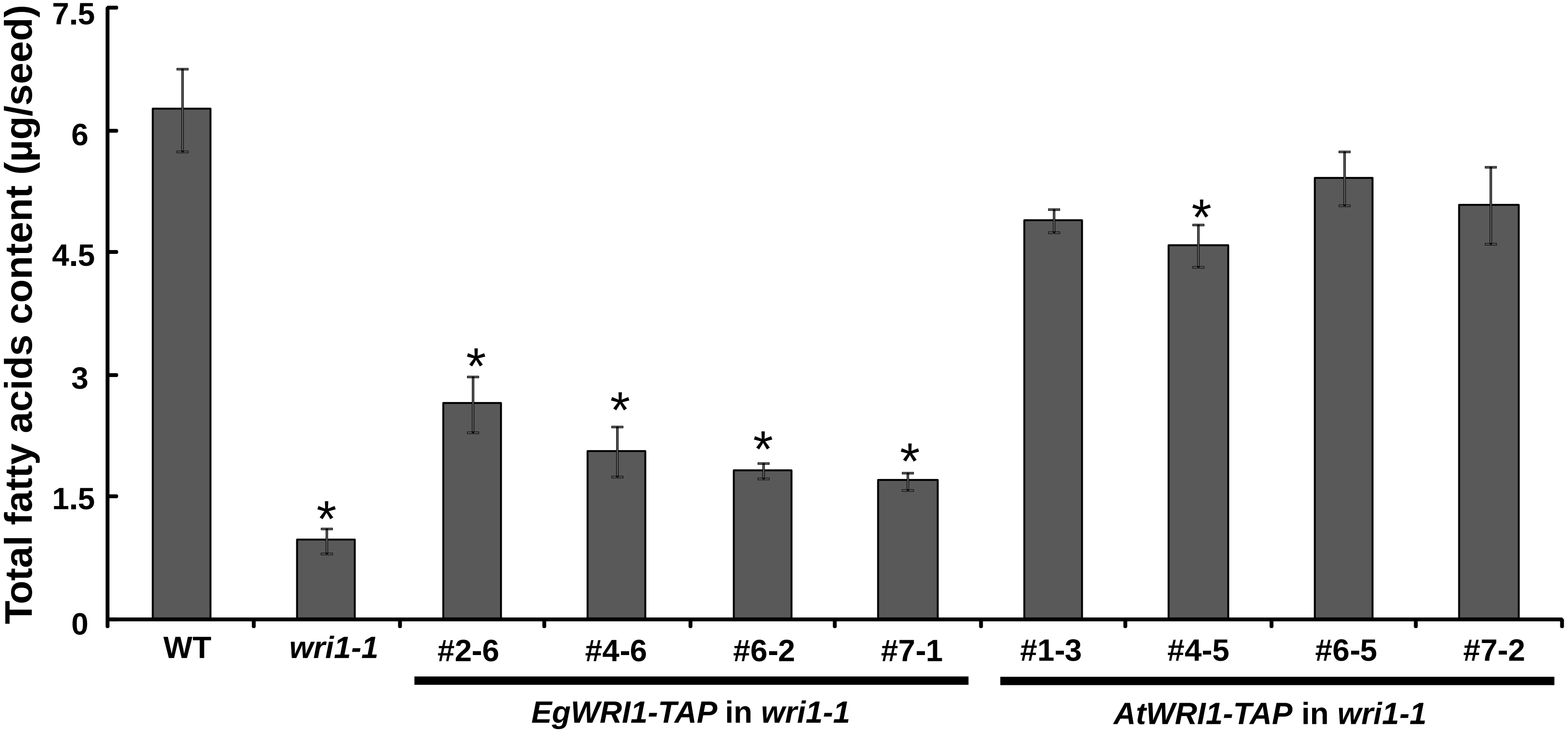

Figure S2. C-terminal TAP-tagged *AtWRI1* rescues the reduced oil phenotype of *wri1-1* mutant. However, C-terminal TAP-tagged *EgWRI1* fails to rescue the reduced oil of *wri1-1.* Results are means ± SE (n = 3-4). “*” indicates significant difference ($P<0.05$, t-test) between WT and other plants.
