# Supplementary material for: WRINKLED1, A Ubiquitous Regulator in Oil Accumulating Tissues from Arabidopsis Embryos to Oil Palm Mesocarp
Source: PLoS One. 2013 Jul 26;8(7):e68887. doi: 10.1371/journal.pone.0068887 (PMC3724841; doi:10.1371/journal.pone.0068887)
Supplement: Figure S3 — Four independent transgenic lines overexpressing EgWRI1-TAP (#2-6, #4-6, #6-2, and #7-1 respectively; from left to right) and four independent transgenic lines expressing AtWRI1-TAP (#1-3, #4-5, #6-5, and #7-2, respectively; from left to right) are shown below. Results are shown as means ± SE (n = 3-4). (PDF) [file pone.0068887.s003.pdf]

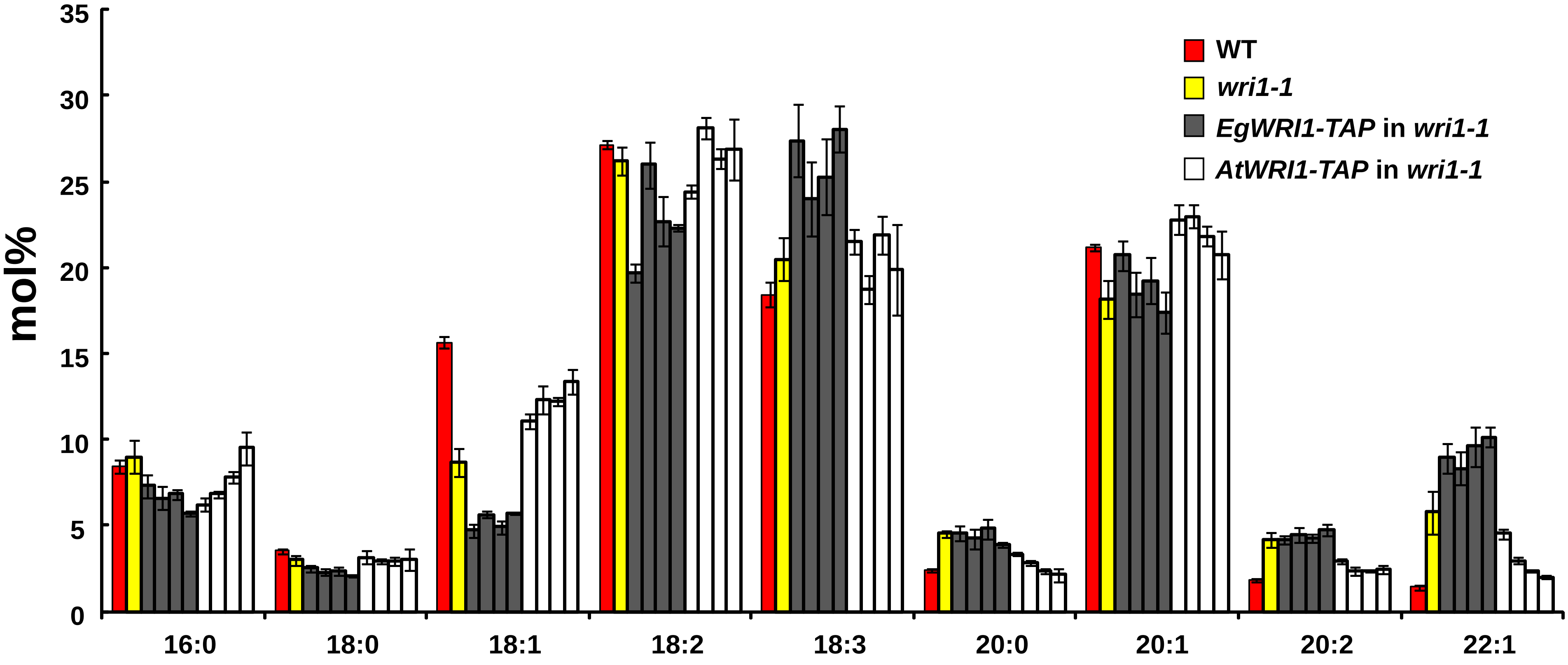

Figure S3. Profiles of seed fatty acid composition of WT, *wri1-1* and *wri1-1* overexpressing *EgWRI1-TAP* and *AtWRI1-TAP*. Four independent transgenic lines overexpressing *EgWRI1-TAP* (#2-6, #4-6, #6-2, and #7-1 respectively; from left to right) and four independent transgenic lines expressing *AtWRI1-TAP* (#1-3,#4-5,#6-5, and #7-2, respectively; from left to right) are shown below. Results are shown as means ± SE (n = 3-4).
