# Supplementary material for: WRINKLED1, A Ubiquitous Regulator in Oil Accumulating Tissues from Arabidopsis Embryos to Oil Palm Mesocarp
Source: PLoS One. 2013 Jul 26;8(7):e68887. doi: 10.1371/journal.pone.0068887 (PMC3724841; doi:10.1371/journal.pone.0068887)
Supplement: Figure S4 — A) C-TAP-tagged EgWRI1 and AtWRI1 were both able to complement the reduced germination of wri1-1 seeds. Results are shown as means ± SE (n =3-4). The seeds germination of EgWRI1-TAP transgenic lines compared to WT were not significantly different (P > 0.05, t-test). B) Transgenic wri1-1 plants expressing AtWRI1-TAP or EgWRI1-TAP. Plants were grown in medium without the addition of sucrose. (PDF) [file pone.0068887.s004.pdf]

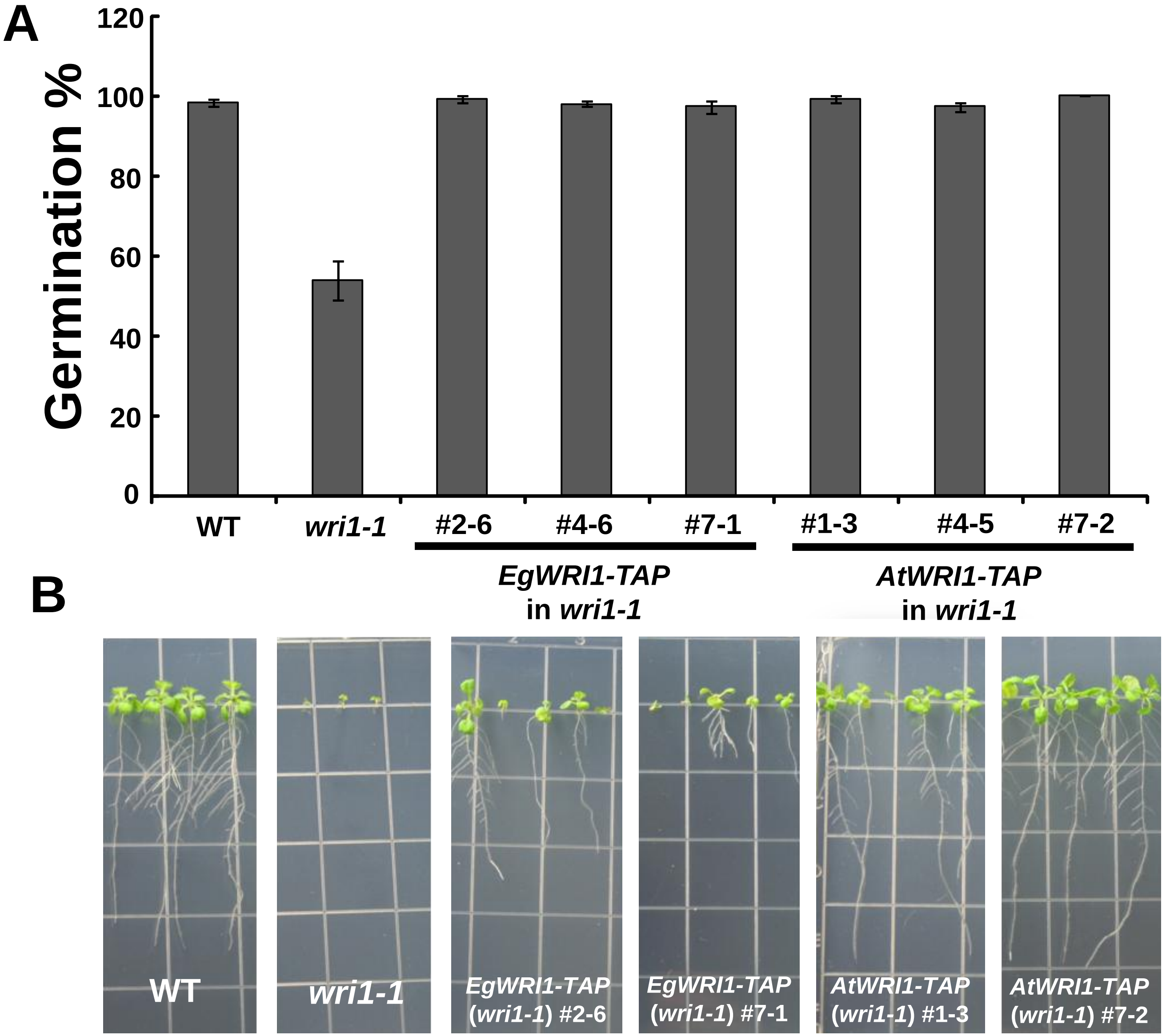

Figure S4. Phenotypes of *wri1-1* plants expressing *EgWRI1-TAP* or *AtWRI1-TAP*. **A)** C-TAP-tagged *EgWRI1* and *AtWRI1* were both able to complement the reduced germination of *wri1-1* seeds. Results are shown as means ± SE (n =3-4). The seeds germination of *EgWRI1-TAP* transgenic lines compared to WT were not significantly different (P > 0.05, t-test). **B)**Transgenic *wri1-1* plants expressing *AtWRI1-TAP* or *EgWRI1-TAP*. Plants were grown in medium without the addition of sucrose.
