# Supplementary material for: WRINKLED1, A Ubiquitous Regulator in Oil Accumulating Tissues from Arabidopsis Embryos to Oil Palm Mesocarp
Source: PLoS One. 2013 Jul 26;8(7):e68887. doi: 10.1371/journal.pone.0068887 (PMC3724841; doi:10.1371/journal.pone.0068887)
Supplement: Figure S5 — A) Alignment of section of AP2 domain of WRI1 orthologs from B. napus, maize and oil palm indicating the most conserved amino acids across different WRI1s. Amino acids “VYL” (highlighted by a red box), are highly conserved in plant WRI1s. B) Alignment of WRI1 ortholog amino acid sequences predicted from genome sequencing information at Phytozome (http://www.phytozome.org/). Of the 34 predicted WRI1-like protein sequences, amino acid sequence “VYL” (highlighted by red box) is missing in 13. The locus IDs of predicted WRI1 orthologs are as follows. M.esculenta (cassava4.1_029667m.g); R.communis (30069.t000002); L.usitatissimum (Lus10008939.g); P.trichocarpa (Potri.008G011900); P.vulgaris (Phvul.011G187400); G.max (Glyma08g24420); C.sativus (Cucsa.282940); P.persica (ppa023152m.g); M.domestica (MDP0000186581); F.vesca (gene00377-v1.0-hybrid); A.thaliana (AT3G543200; A . lyrata (485830); C . rubella (Carubv10018845m.g); B . rapa (Bra007066); T. halophila (Thhalv10010394m.g); C. papaya (evm. TU. supercontig_54.28); G . raimondii (Gorai.011G225700); T . cacao (Thecc1EG044588); C. sinensis (orange1.1g036423m.g); C . clementina (Ciclev10003896m.g); E . grandis (Eucgr.J00316); V. vinifera (GSVIVG01020066001); S. tuberosum (PGSC0003DMG400027502); S . lycopersicum (Solyc01g096860.1); M . guttatus (mgv1a007319m.g); A . coerulea (Aquca_016_00348); S. bicolor (Sb02g025080); Z. mays (GRMZM2G141219); S . italica (Si030129m.g); P . virgatum (Pavirv00024549m.g); O. sativa (LOC_Os11g03540); B . distachyon (Bradi4g30617); S . moellendorffii (85823); P . patens (Pp1s32_65V6). (PDF) [file pone.0068887.s005.pdf]

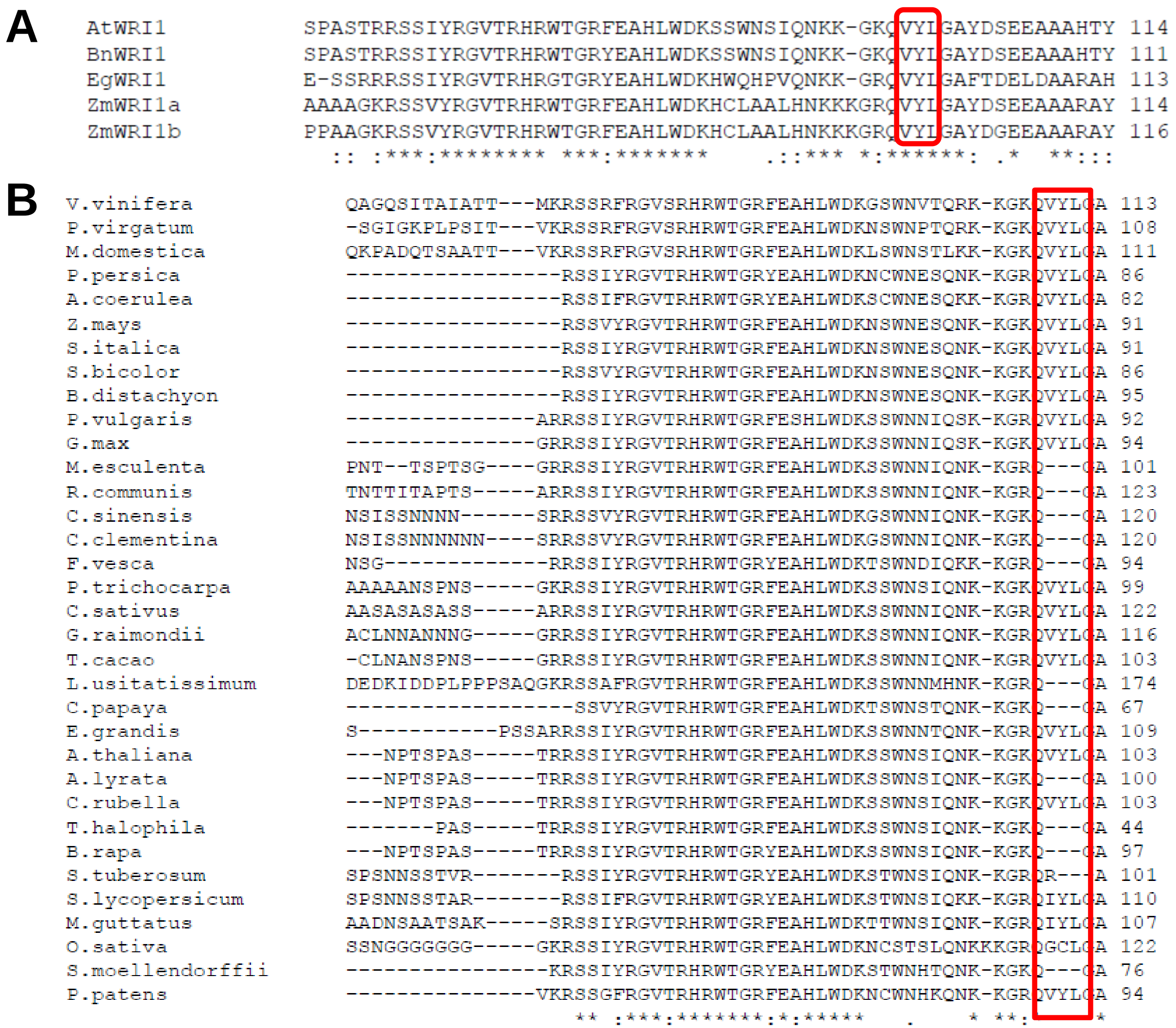

Figure S5. Alignment of WRI1 orthologs. **A**) Alignment of section of AP2 domain of WRI1 orthologs from *B. napus*, maize and oil palm indicating the most conserved amino acids across different WRI1s. Amino acids "VYL" (highlighted by a red box), are highly conserved in plant WRI1s. **B**) Alignment of WRI1 ortholog amino acid sequences predicted from genome sequencing information at Phytozome (http://www.phytozome.org). Of the 34 predicted WRI1-like protein sequences, amino acid sequence "VYL" (highlighted by red box) is missing in 13. The locus IDs of predicted WRI1 orthologs are as follows. *M.esculenta* (cassava4.1_029667m.g); *R.communis* (30069.t000002); *L.usitatissimum* (Lus10008939.g); *P.trichocarpa* (Potri.008G011900); *P.vulgaris* (Phvul.011G187400); *G.max* (Glyma08g24420); *C.sativus* (Cucsa.282940); *P.persica* (ppa023152m.g); *M.domestica* (MDP0000186581); *F.vesca* (gene00377-v1.0-hybrid); *A.thaliana* (AT3G543200; *A.lyrata* (485830); *C.rubella* (Carubv10018845m.g); *B.rapa* (Bra007066); *T.halophila* (Thhalv10010394m.g); *C.papaya* (evm.TU.supercontig_54.28); *G.raimondii* (Gorai.011G225700); *T.cacao* (Thecc1EG044588); *C.sinensis* (orange1.1g036423m.g); *C.clementina* (Ciclev10003896m.g); *E.grandis* (Eucgr.J00316); *V.vinifera* (GSVIVG01020066001); *S.tuberosum* (PGSC0003DMG400027502); *S.lycopersicum* (Solyc01g096860.1); *M.guttatus* (mgv1a007319m.g); *A.coerulea* (Aquca_016_00348); *S.bicolor* (Sb02g025080); *Z.mays* (GRMZM2G141219); *S.italica* (Si030129m.g); *P.virgatum* (Pavirv00024549m.g); *O.sativa* (LOC_Os11g03540); *B.distachyon* (Bradi4g30617); *S.moellendorffii* (85823); *P.patens* (Pp1s32_65V6).
