# Supplementary material for: WRINKLED1, A Ubiquitous Regulator in Oil Accumulating Tissues from Arabidopsis Embryos to Oil Palm Mesocarp
Source: PLoS One. 2013 Jul 26;8(7):e68887. doi: 10.1371/journal.pone.0068887 (PMC3724841; doi:10.1371/journal.pone.0068887)
Supplement: Figure S6 — A) Two pairs of PCR primers were designed, which cover the region of AtWRI1 exon3 and last intron, respectively. The length of PCR product with primers set 1 (FW1+RV1) is 157bp (splice form 1 and 3) and 148bp (splice form2), respectively. The length of PCR product with primer set 2 (FW2+RV2) is 167bp (splice form2; last intron is spliced out) and 269bp (splice form 2 and 3; last intron is not spliced out), respectively. AtWRI1 transcript accumulation in Arabidopsis plants was analyzed by semi-quantitative RT-PCR, with primers set 1 (B) and primers set 2 (C), respectively. Samples with even numbers were grown in growth medium containing 3% sucrose. Samples with odd numbers were grown in growth medium without the addition of sucrose. Arabidopsis seedlings are 3- (sample 1 & 2), 4- (sample 3 & 4), 5- (sample 5 & 6), 6- (sample 7 & 8), 7- (sample 9 & 10), 8-(sample 11 & 12), and 9- (sample 13 & 14) day-old, respectively. (PDF) [file pone.0068887.s006.pdf]

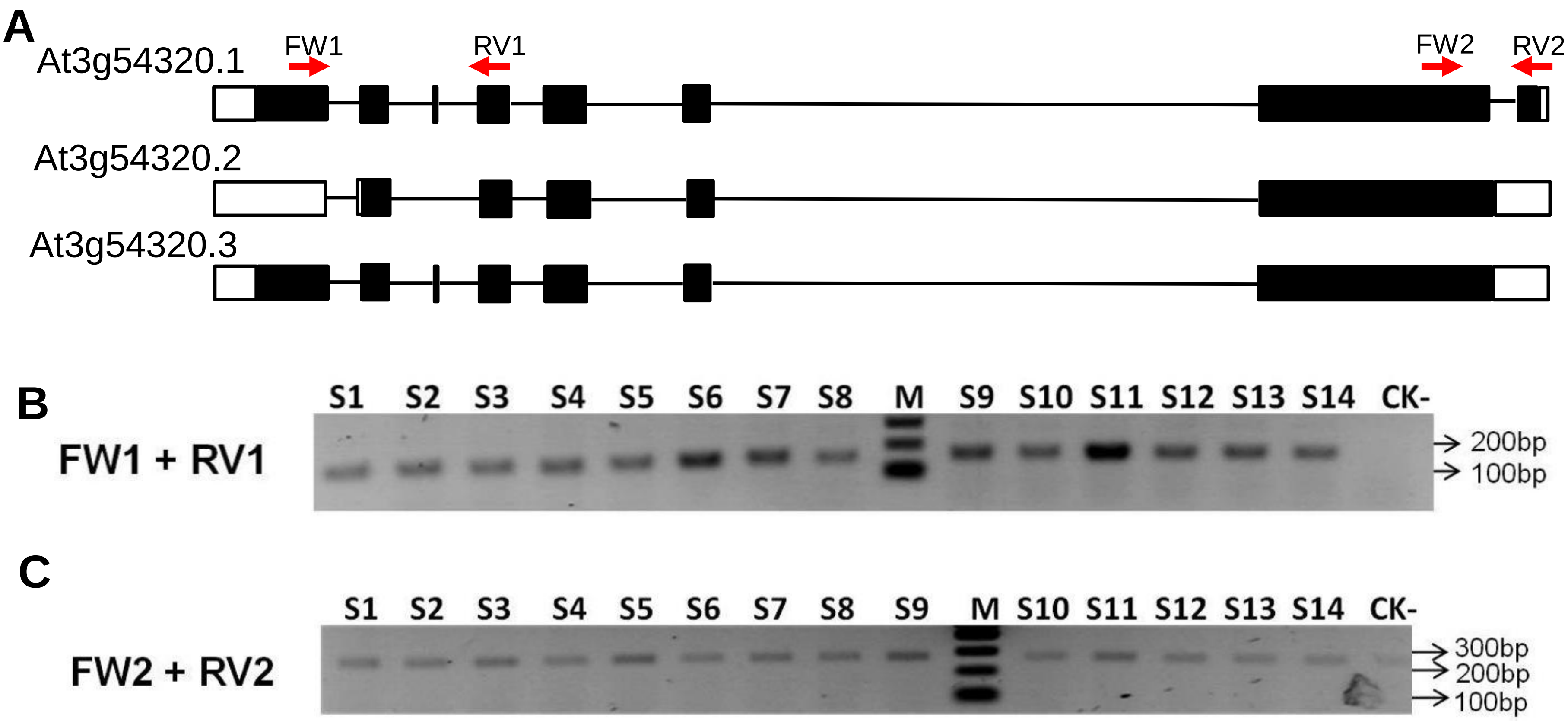

Figure S6. Analysis of *AtWRI1* splice forms by RT-PCR. **A)** Two pairs of PCR primers were designed, which cover the region of *AtWRI1* exon3 and last intron, respectively. The length of PCR product with primers set 1 (FW1+RV1) is 157bp (splice form 1 and 3) and 148bp (splice form2), respectively. The length of PCR product with primer set 2 (FW2+RV2) is 167bp (splice form2; last intron is spliced out) and 269bp (splice form 2 and 3; last intron is not spliced out), respectively. *AtWRI1* transcript accumulation in Arabidopsis plants was analyzed by semi-quantitative RT-PCR, with primers set 1 (**B**) and primers set 2 (**C**), respectively. Samples with even numbers were grown in growth medium containing 3% sucrose. Samples with odd numbers were grown in growth medium without the addition of sucrose. Arabidopsis seedlings are 3- (sample 1&2), 4- (sample 3&4), 5- (sample 5&6), 6- (sample 7&8), 7- (sample 9&10), 8- (sample 11&12), and 9- (sample 13&14) day-old, respectively.
