# Supplementary material for: WRINKLED1, A Ubiquitous Regulator in Oil Accumulating Tissues from Arabidopsis Embryos to Oil Palm Mesocarp
Source: PLoS One. 2013 Jul 26;8(7):e68887. doi: 10.1371/journal.pone.0068887 (PMC3724841; doi:10.1371/journal.pone.0068887)
Supplement: Figure S7 — We analyzed >100 million Illumina reads from developing Arabidopsis seeds. Of these, ~10,000 or 1% mapped to the AtWRI1 gene. 500 of these reads mapped to the genome region that included the 9 bp exon 3. A subset of the reads is presented based on visualization of alignment by GBrowse (http://www.gbrowse.org). No reads were detected that lacked exon 3. Similar analysis of 3’ sequences indicated only splice form 3 was represented. (PDF) [file pone.0068887.s007.pdf]

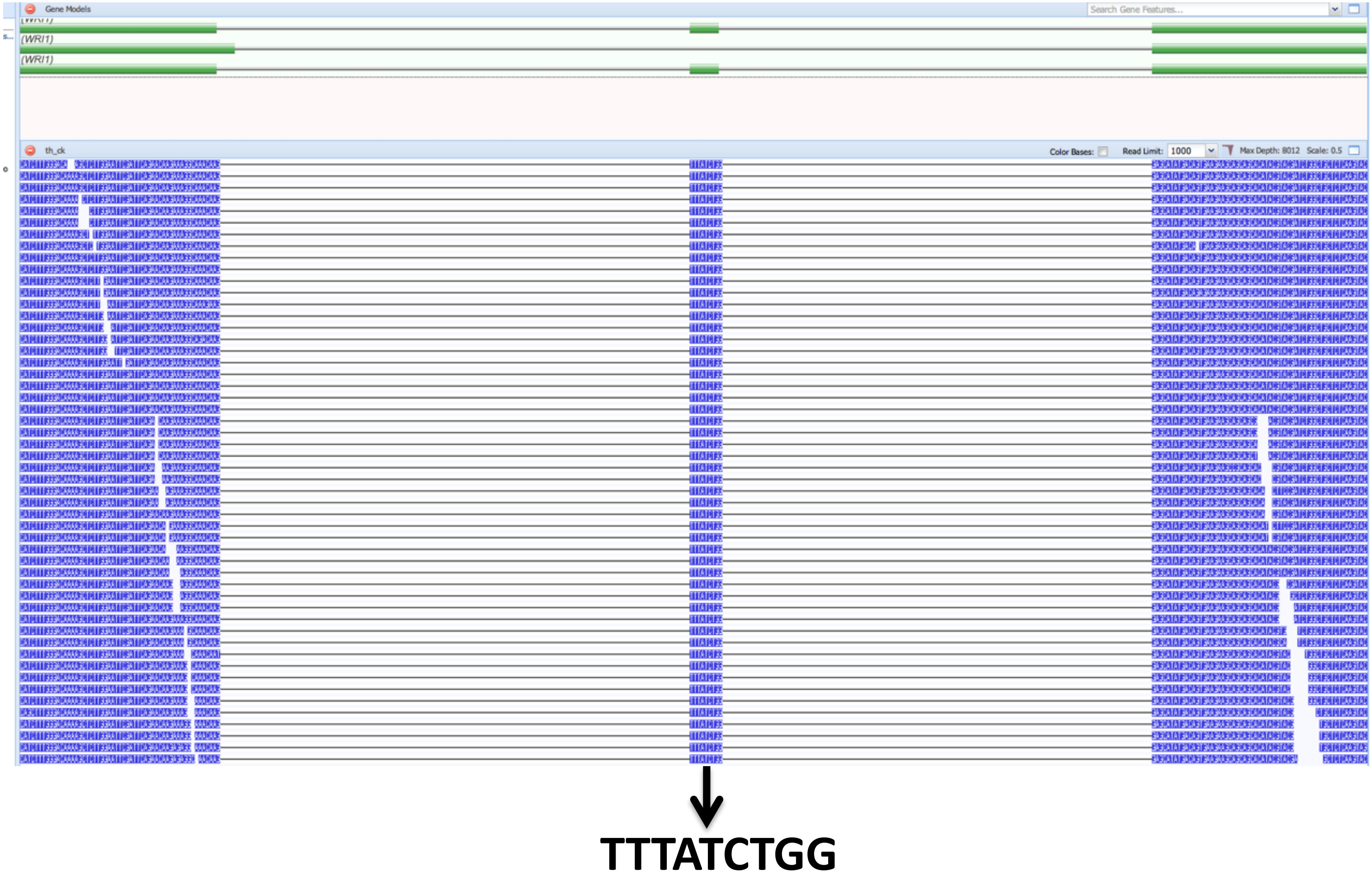

Figure S7. Alignment of Illumina RNASeq reads from mRNA of developing seeds of Arabidopsis. We analyzed >100 million Illumina reads from developing Arabidopsis seeds. Of these, ~10,000 or 1% mapped to the *AtWRI1* gene. 500 of these reads mapped to the genome region that included the 9 bp exon 3. A subset of the reads is presented based on visualization of alignment by GBrowse (www.gbrowse.org). No reads were detected that lacked exon 3. Similar analysis of 3' sequences indicated only splice form 3 was represented.
