# Supplementary material for: WRINKLED1, A Ubiquitous Regulator in Oil Accumulating Tissues from Arabidopsis Embryos to Oil Palm Mesocarp
Source: PLoS One. 2013 Jul 26;8(7):e68887. doi: 10.1371/journal.pone.0068887 (PMC3724841; doi:10.1371/journal.pone.0068887)
Supplement: Figure S8 — A) A 69 nucleotide sequence was designed to distinguish AtWRI1 splice form 1 and 3 from form 2. The sequence includes the nine nucleotides that encode “VYL” (highlighted in green), together with 30 nucleotides 5’ and 3’ flanking sequences. B) Position of diagnostic search sequence is highlighted by red boxes in the picture of alignment of predicted partial cDNAs of three AtWRI1 alternative splice forms. (PDF) [file pone.0068887.s008.pdf]

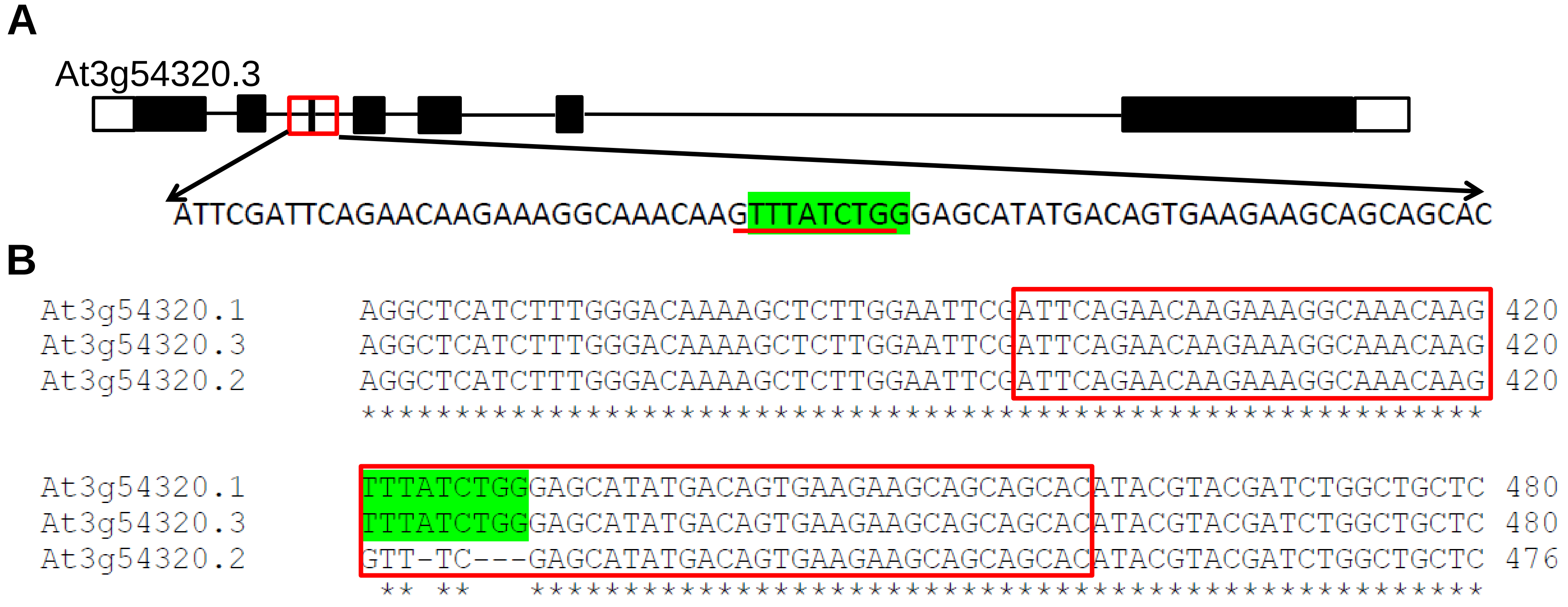

Figure S8. Diagnostic search sequence for exon 3. **A)** A 69 nucleotide sequence was designed to distinguish *AtWRI1* splice form 1 and 3 from form 2. The sequence includes the nine nucleotides that encode "VYL" (highlighted in green), together with 30 nucleotides 5' and 3' flanking sequences. **B)** Position of diagnostic search sequence is highlighted by red boxes in the picture of alignment of predicted partial cDNAs of three *AtWRI1* alternative splice forms.
