# Supplementary material for: WRINKLED1, A Ubiquitous Regulator in Oil Accumulating Tissues from Arabidopsis Embryos to Oil Palm Mesocarp
Source: PLoS One. 2013 Jul 26;8(7):e68887. doi: 10.1371/journal.pone.0068887 (PMC3724841; doi:10.1371/journal.pone.0068887)
Supplement: Figure S9 — A) The flanking sequence upstream from the last intron which is spliced out in AtWRI1 splice form 1 was chosen as a diagnostic search sequence for the 3’ end splice form search. B) Position of the diagnostic search sequence for WRI1 3’ end is highlighted by red boxes in alignment of predicted partial cDNAs of three AtWRI1 alternative splice forms. (PDF) [file pone.0068887.s009.pdf]

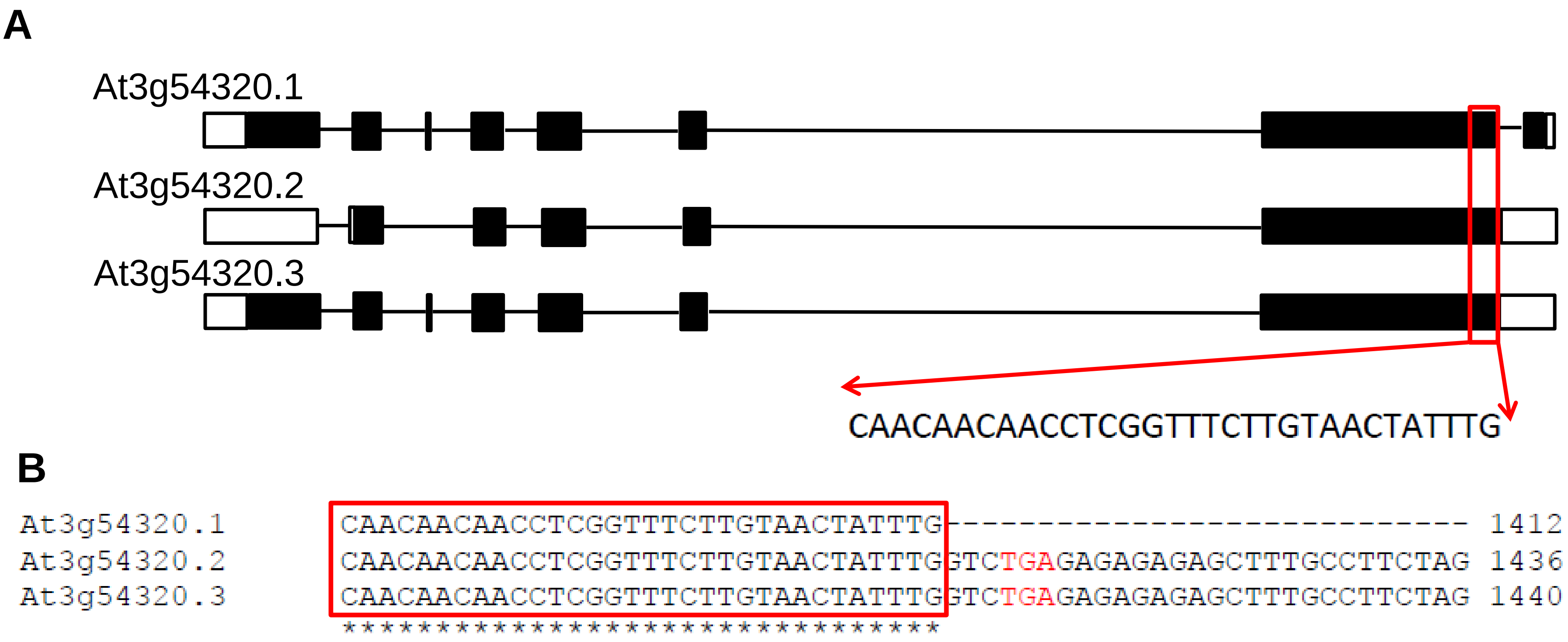

Figure S9. Diagnostic search sequence for *AtWRI1* 3' end. **A)** The flanking sequence upstream from the last intron which is spliced out in *AtWRI1* splice form 1 was chosen as a diagnostic search sequence for the 3' end splice form search. **B)** Position of the diagnostic search sequence for *WRI1* 3' end is highlighted by red boxes in alignment of predicted partial cDNAs of three *AtWRI1* alternative splice forms.
