# Supplementary material for: WRINKLED1, A Ubiquitous Regulator in Oil Accumulating Tissues from Arabidopsis Embryos to Oil Palm Mesocarp
Source: PLoS One. 2013 Jul 26;8(7):e68887. doi: 10.1371/journal.pone.0068887 (PMC3724841; doi:10.1371/journal.pone.0068887)
Supplement: Figure S10 — (PDF) [file pone.0068887.s010.pdf]

ATGACTCTTATGAAGAACTCTCCTCCTTCTACACCTTTGCCTCCAATTTCTCCTAGTTCTTCTGCTT
CTCCAAGTTCTTACGCTCCACTTTCTTCTCCAAACATGATCCCTCTCAACAAGTGCAAGAAGTCT
AAGCCTAAGCACAAGAAAGCTAAGAACTCTGATGAGTCATCTAGAAGAAGGTCATCTATCTACAG
GGGAGTGACTAGACATAGAGGAACTGGAAGATACGAGGCTCATCTTTGGGATAAGCACTGGCAA
CATCCTGTTCAAAACAAGAAGGGAAGGCAGGTTTACCTTGGAGCTTTTACTGATGAGCTTGATGC
TGCTAGGGCTCATGATCTTGCTGCTCTTAAACTTTGGGGACCTGAGACTATCCTCAATTTTCCTGT
TGAGATGTACAGGGAAGAGTACAAAGAAATGCAGACCATGTCTAAAGAAGAGGTTCTCGCTTCTG
TGAGAAGAAGATCTAACGGATTCGCTAGAGGAACCTCTAAGTATAGAGGTGTGGCTAGACATCAC
AAGAATGGAAGATGGGAGGCTAGACTTTCTCAGGATGTTGGATGCAAGTACATCTACCTTGGAAC
TTACGCTACCCAAGAAGAGGCTGCTCAGGCTTATGATTTGGCTGCTTTGGTTCATAAGGGACCTA
ACATCGTGACCAACTTTGCTTCTTCTGTGTACAAGCACAGACTCCAACCTTTCATGCAGCTTTTG
GTTAAGCCTGAAACTGAACCTGCTCAAGAGGATCTTGGAGTTCTTCAAATGGAAGCTACCGAGA
CTATCGATCAGACTATGCCTAACTACGATCTCCCTGAGATCTCTTGGACCTTCGATATCGATCATG
ATCTTGGAGCTTACCCTCTTCTCGATGTTCCTATTGAGGATGATCAGCACGATATCCTCAACGATC
TTAACTTCGAGGGAAACATCGAGCATCTCTTCGAAGAATTCGAGACTTTTGGAGGAAACGAGTCT
GGATCTGATGGATTTTCTGCTTCTAAGGGAGCTTGA

Figure S10: Nucleotide sequence of synthetic *EgWRI1*.
