# Supplementary material for: WRINKLED1, A Ubiquitous Regulator in Oil Accumulating Tissues from Arabidopsis Embryos to Oil Palm Mesocarp
Source: PLoS One. 2013 Jul 26;8(7):e68887. doi: 10.1371/journal.pone.0068887 (PMC3724841; doi:10.1371/journal.pone.0068887)
Supplement: Table S1 — (PDF) [file pone.0068887.s011.pdf]

Primer name	Sequence 5' to 3'	Purpose
EgWRI1-FW	5'-ACTGGATCCGGACAATGACTCTTATG-3'	Forward primer for cloning full length of EgWRI1
EgWRI1-RV1	5'-TATCTCGAGTGAGCTCCCTTAGA-3'	Reverse primer for cloning full length of EgWRI1 without stop codon
EgWRI1-RV2	5'-TATCTCGAGTCAAGCTCCCTTAGA-3'	Reverse primer for cloning full length of EgWRI1 with stop codon
AtWRI1-FW	5'-AATGGATCCGGACAATGAAGAAGCGCTTA-3'	Forward primer for cloning full length of AtWRI1
AtWRI1-RV1	5'-TCCCTCGAGTCAGACCAAATAGTT-3'	Reverse primer for cloning full length of AtWRI1 with stop codon
AtWRI1-RV2	5'-TCCCTCGAGTGGACCAAATAGTT-3'	Reverse primer for cloning full length of AtWRI1 without stop codon
AtWRI1$^{V99A/Y100A/L101A}$-RV	5'-GTCATATGCTCCTGCTGCTGCTTGTTTGCCTTTCTTG-3'	Reverse primer for amplifying site-specific mutagenesis AtWRI1$^{V99A/Y100A/L101A}$
AtWRI1$^{V99A/Y100A/L101A}$-FW	5'-AGAAAGGCAAACAAGCAGCAGCAGGAGCATATGACAGT-3'	Forward primer for amplifying site-specific mutagenesis AtWRI1$^{V99A/Y100A/L101A}$
AtWRI1^{V99D}-RV	5'-TCATATGCTCCCAGATAATCTTGTTTG-3'	Reverse primer for amplifying site-specific mutagenesis AtWRI1^{V99D}
AtWRI1^{V99D}-FW	5'-CAAACAAGATTATCTGGGAGCATATGA-3'	Forward primer for amplifying site-specific mutagenesis AtWRI1^{V99D}
AtWRI1^{Y100C}-RV	5'-TCATATGCTCCCAGACAAACTTGTTTG-3'	Reverse primer for amplifying site-specific mutagenesis AtWRI1^{Y100C}
AtWRI1^{Y100C}-FW	5'-CAAACAAGTTTGTCTGGGAGCATATGA-3'	Forward primer for amplifying site-specific mutagenesis AtWRI1^{Y100C}
AtWRI1^{L101Q}-RV	5'-TCATATGCTCCTTGATAAACTTGTTTG-3'	Reverse primer for amplifying site-specific mutagenesis AtWRI1^{L101Q}
AtWRI1^{L101Q}-FW	5'-CAAACAAGTTTATCAAGGAGCATATGA-3'	Forward primer for amplifying site-specific mutagenesis AtWRI1^{L101Q}
35S-FW	5'-ATCTCTCTGCCGACAGTGGT-3'	CaMV 35S promoter forward primer
FW1	5'-CCCGACGCAGCTCTATCTAC-3'	Forward primer for amplifying the small exon region of AtWRI1
RV1	5'-TGTGCTGCTGCTTCTTCACT-3'	Reverse primer for amplifying the small exon region of AtWRI1
FW2	5'-CACGAGTGCTTGAACTTGGA-3'	Forward primer for amplifying the 3' end region of AtWRI1
RV2	5'-CCAACGAACAAGCCCTGAAA-3'	Reverse primer for amplifying the 3' end region of AtWRI1

Table S1. Primers used in this study.
